# A case study of a real-time internet of things system for site-specific potato crop management in El-Salhia Area-Egypt

**DOI:** 10.1038/s41598-022-22690-3

**Published:** 2022-11-03

**Authors:** Basma M. Mohammad EL-Basioni, Elsayed Said Mohamed, AA. Belal, Mohamed E. M. Jalhoum, Sherine M. Abd EL-Kader, Mohamed B. Zahran

**Affiliations:** 1 Electronics Research Institute (ERI), Cairo, Egypt; 2 National Authority for Remote Sensing and Space Sciences (NARSS), Cairo, Egypt

**Keywords:** Electrical and electronic engineering, Environmental sciences

## Abstract

The site-specific management is the technology that considers the natural variability within the same field of factors related to crop growth to improve its management practices such that the agricultural treatments are varied for field's small production zones saving resources and environment, and improving crop quality and size. Since site-specific decisions are not far from the Fourth Industrial Revolution and the concept of processes automation, this work addresses improving the process of spatial variability analysis and thus supporting management decisions by developing a system—entitled EGYPADS—based on the Internet of Things and its enabling technologies. EGYPADS automates data collection, zones delineation according to their land suitability evaluation, and maps generation. The paper addresses a case study of potato crop in a specific area in Egypt, El-Salhia, in which eighty-five sites were chosen as main dataset for the modeling process during different stages of crop growth. Three management zones were recognized of the selected field based on the differentiation in their land suitability characteristics, representing about 5%, 65%, and 30% of the total area, respectively. The structure, screens, and services of EGYPADS are described in this paper. EGYPADS offered services include: management zones delineation using absolute and virtual coordinates, Land Suitability Assessment (LSA), data entry from field in real-time as well as from excel sheets, saving maps in suitable format for variable rate application, real-time and historical data processing, centralized management, and flexible formulation of events and related actions. The implementation of EGYPADS was verified. The system dynamically produces non-contiguous isobands, each representing a specific range of parameter values, and can be properly exported for use by other programs or smart machinery. It was proven that EGYPADS supports more than one land with different geometry, area, location, and number of nodes. EGYPADS was compared with the traditional LSA method, and was found to produce similar management zones.

## Introduction

The agricultural sector contributes about 16% of the Egyptian Gross Domestic Product (GDP), in addition it provides about 30% of job opportunities for youth^1^. Therefore, the interest in developing the agricultural sector is very important for growing the Egyptian economy^2,3^. There are many challenges that hinder agricultural production in Egypt, for example, the reduction of agricultural ownership, soil loosed due to increasing urban sprawl, etc.^4–6^, where it has been observed that approximately 50% of the farms in the Nile Delta have an area less than one acre^7^. Other challenges that are facing the agricultural production in Egypt include: reliance of most farmers on traditional agricultural methods, climate change, deficiency of irrigation water, determining the proper time for fertilization and pest resistance, etc. Most of these challenges may be due to a lack of understanding of the spatial distribution of soil characteristics, LSA and their relationship to the crop^8^.

The potato is considered one of the essential crops for most people around the world, the total global production of about 360 million metric meters. Annual production of fruits and vegetables in Egypt was about 35 million tons in 2018 according to Food and Agriculture Organization (FAO), in addition potato is one of the highest production crops among vegetables. Statistics indicate that Egyptian potato exports reached 205 million US dollars in 2018. Egypt exported more than 724,200 thousand tons of potatoes in 2018 and became the fifth largest exporter of potatoes, mainly to Russia and the European Union^9^. Potato crop is not far from the challenges that face the Egyptian agricultural production; thus due to its extreme importance with the great challenge and problems facing its production and export, the study in this paper was chosen to mainly revolve around the potato crop and specifically addresses the agricultural practice related to assessing the suitability of the land for its production.

During the past two decades, Precision Agriculture (PA) techniques have proven successful in improving the farm management and contributing to addressing the agricultural problems, on the other hand increasing the agricultural profitability benefit based on more accurate information on the agricultural processes^10,11^. Reducing the cost of farming while increasing productivity is often seen as a win-win strategy for managing resources and sustainable agricultural production^12^. During the agricultural process, farmers need help in making the right decision to manage their farm during the crop growth stages.

PA allows monitoring and tracking plant growth in different stages, monitoring the change in climate factors, as well as monitoring the change in soil moisture status to determine the appropriate time for irrigation and irrigation scheme^13–15^. Soil type, salinity, moisture, pH, depth, and drainage characteristics, as well as their distribution influence farm production, thus, understanding the dynamic availability of soil resources is a key to soil management^14,16–18^. The precision control of soil resources within the farm can either ensure sustainability or enhance the cropping intensity with appropriate interventions.

Since the appearance of PA concept in the early 1980s, a lot of related technologies and methods^19^ have been developed and evolved over time. Perhaps the most prominent technologies used in PA, which clearly appeared in its proposed solutions, are: the remote sensing which is a geospatial technology used for monitoring the physical characteristics of an agricultural field on the earth from a distance by acquiring its emitted or reflected electromagnetic radiation via sensors deployed on satellites or aircrafts; the Global Positioning System (GPS) which is a satellite-based radio-navigation system in which the agricultural data collection devices are equipped with GPS receiver calculates its distance from at least four satellites via the time taken by radio signals to propagate from each one, and from this it calculates the device real location on the earth; the Geographic Information Systems (GIS) which is the software tool used to manage and analyze the acquired spatial data to deduce important implicit conclusion^20–23^; spatial analysis and mapping techniques; Decision Support System (DSS) which represents a software technique utilizes a provided data to produce conclusions and guidance supports decision-makers in specific agricultural practice.

The DSS for the agricultural management is quite complex; it needs specific information about agricultural processes, such as climate conditions, many variables affect crop growth where each crop requires a different optimum value for growth^20,21^. The success of DSS decisions in managing the agricultural process depends on the quality of information about the stages of growth of agricultural crops, while the traditional data acquisition technologies such as remote sensing and the GPS-equipped handheld data collection devices suffer from some limitations affect its accuracy such as the low temporal and spatial resolution, limited variety of available soil and plant data, and impossibility of implementing automation. Therefore, the recent technologies Wireless Sensor Network (WSN) and Unmanned Aerial Vehicle (UAV) are introduced as PA-enabling technologies for data acquisition solving a lot of their past technologies limitations and problems^22–25^.

A WSN comprises wireless nodes contain sensors with variety of sensing modalities; these sensor nodes are deployed in the field to sense and send its related real-time data continuously to the software system where it is stored and utilized. These devices also can receive commands from the software application control its integrated actuators achieving a closed- or open-loop control system for the farm. The UAVs or the agriculture drones can be regarded as a sensor/actuator node has the ability to fly at low altitude from the crop which aid in producing high spatial resolution imagery and detect tiny crop details that can be used for example to distinguish between crop diseases. Thus, UAVs can be used to produce accurate field mapping, along with other applications such as crop spraying.

When talking about the recent PA systems which is based on WSN and emerging information technology, the IoT technology should be a key component in such PA systems^26^. It enables the remote access of the deployed sensing devices on the field which adds flexibility in the system usage and increases its scalability. A complete IoT system is composed of three tiers: the things tier, the IoT connection tier, and the application tier. The things tier includes the IoT devices and the monitored/controlled things. The IoT device is an embedded device has support for internet connectivity; it can be connected to the thing it monitors/controls or a standalone device; it can be a sensor node or a base station just works as forwarder to the data of a WSN through the Internet. The IoT connection tier is implemented by the IoT messaging protocols which define the rules, formats and functions for transferring messages between IoT clients over the Internet. The application tier contains the back- and front-end services that implement the functions of a specific IoT use case and represent the real benefit of the collected IoT data.

The proposed work in this paper belongs to these recent PA systems. EGYPADS can be regarded as an IoT-enabled PA application which is a software system does PA function(s) and equipped to receive data directly from the field devices, on the other hand, it can be viewed as an IoT system dedicated to a specific use case related to the agriculture field concentrates in its current version with the implementation of the second and third IoT tiers.

As an IoT use case, the case in question is the automation of site-specific management and LSA processes for supporting potato crop cultivation management decisions. As a PA software platform it exploits the underlying technologies to add more improved smart agriculture functions and services.

As mentioned earlier EGYPADS implements mainly the second and third IoT tiers. The application tier is web-based controlled by a JavaScript back-end application manages the database, data processing and analysis, web hosting, communication with the front-end, and the IoT communication tier. The IoT tier is implemented using a publish-subscribe messaging pattern^27^ to support asynchronous broadcasting, event-driven operations, and near real-time communication; and generally reaching decoupled components in time, space, and synchronization, thus increasing the system maintainability, reliability, and scalability. The parameters used in the analysis are related to chemical, physical, and fertility characteristics of the soil, as well as some parameters related to weather conditions and plant characteristics. The EGYPADS front-end allows system users to access the system services which are: Spatial Analysis (Mapping Service) using absolute and virtual coordinates; Real-time Data Visualization which includes Climate and Plant Parameters Visualization, Soil Parameters Visualization, and Nodes Status Monitoring Panel; Historical Data Visualization, Events and Actions Settings, and Administrative Settings.

Due to its modularity and by selecting a lightweight messaging protocol suitable for major embedded and non-embedded platforms, EGYPADS exposes API to connect any IoT device by small settings to its IoT client software and the format of the IoT message payload. The current version of EGYPADS also considers a real implementation of the things tier but without full support for the system abilities; it is used only for testing and verification purposes. This tier contains a WSN comprises a base station and sensor nodes forming star topology.

A complete LSA process for potato crop was conducted in an agriculture land in El-Salhia region using the traditional method currently in use, which entails frequent field visits. The conducted field trips and analysis helped in determining the pitfalls of the traditional method, highlighting the need to automate this method, reaching the land assessment criteria and model, setting the values of weights and scores, shaping the requirements of the automated process, and putting the specification of its real-time decision support system and required output. These field trips were also needed to collect soil samples for laboratory analysis to determine the values of some parameters of the assessment model that could not be given as direct readings from devices. Thirteen parameters and three thematic indicators were used in the assessment; their values were interpolated for spatial analysis and their maps were produced.

From the foregoing, it is clear that there is a gap between the method currently used for Management Zone (MZ) delineation especially the LSA practice and the technological advances in electronics, communication, and information management that are invading the field of agriculture today. At the same time, the available smart agriculture solutions miss addressing the automation of this practice. The proposed work was keen to fill this gap, therefore its objectives focused on automating the LSA process and generally providing an enhanced spatial analysis functionality backed by IoT capabilities to facilitate the agricultural management practices and obtain highly accurate results by producing such maps with higher temporal resolution using the real-time field data. The research work in this paper describes the details of designing a novel smart agriculture solution that supports LSA process automation, and combines other smart agriculture services not present in any other systems, or at least they do not exist together in one other system, and presents them in a different form and with greater capabilities. The proposed system—EGYPADS -is characterized by:

- Exploiting the new technologies of IoT communication system, web development, database, and digital mapping to automate the construction of MZ maps directly from the data sent by the infield sensing devices.
- This automation would help reduce expenses, time, and effort consumed in repetitive field visits to collect field data that are used in mapping, and it would also help avoid human error in taking or recording readings and increase the temporal accuracy of mapping.
- The spatial modeling and analysis of the phenomena is based on the standard and common methods.
- Producing thematic maps of soil properties such as soil salinity, soil depth, etc., from real-time as well as historical data.
- LSA and construction of soil MZs of any selected farm using real-time as well as historical data.
- Facilitation to construct maps through both methods: the ordinary used method which requires the data collected from the field trips to be entered manually into the application through sheets, and automatically from the data sent by infield devices.
- Ability to export maps in standard formats.
- Facilitation and flexibility to formulate a complete complex event to be tested such that any critical condition can be detected and dealt early.
- Ability to use virtual coordinates for spatial analysis if Global Positioning System (GPS) is not available and the area is not large.
- Ability to register any land having any geometrical shape with the system and facility for remote central monitoring.

This paper explains activities of the field trips; the architecture, requirements, screens, functions, and services of the proposed system; and its functionality verification. The rest of this paper is organized as follows, “Literature review” section reviews the related work, “Materials and methodology” section introduces the spatial modeling and the proposed system architecture. “Results” section shows the results of the system implementation and validation. “Discussion” section is a discussion of the work presented in this paper taking the character of comparison between using the system and using the current used method for spatial analysis. Finally, “Conclusion and future work” section concludes the paper.

## Literature review

A large number of uncountable research and commercial solutions have been proposed for each PA technology highlighted in the previous section or a combination of them. This section will shed the light on some examples of them.

WSN and UAV are used in a lot of applications such as soil sampling, field mapping, tractor guidance, crop scouting, variable rate applications, etc. In^28^, the images taken by three different types of camera (multispectral, thermal, and RGB) loaded on a UAV were exploited for characterizing vineyard spatial variability based on vegetative vigor, water stress and missing plant detection, respectively. A ground station receives the real-time video data from the UAV through a WiFi connection, and it controls it using a duplex transmitter. After performing image processing and some calculations, maps of different indices were elaborated and classification were performed.The system was tested for real lands in clear sky conditions and proved good performance and ability to aid in giving precision irrigation recommendations, but this is not without some flaws such as negative performance indicators with respect to missing plants detection, and lack of a precise monitoring of other descriptive ground parameters related to plant status, soil, microclimate, yield and quality. In addition, it is appropriate for small and medium scale agricultural lands; the monitoring operational time of the UAV is determined to be 15 min.

A huge number of smart farming applications based on WSN were presented in the literature. In^29^, the solution is presented to improve water consumption and at the same time improve crop size by making available the information by which cultivation, harvesting, irrigation, and fertilization can be efficiently scheduled. It monitors temperature, relative humidity, and soil moisture, and formulates a report of them contains also a 10-day weather forecast.

Some of the proposed applications were suitable to only one farm or some adjacent farms, and for small to medium farms scale, a local laptop or personal digital assistant was employed to run the application and monitor the farm. To be able to monitor and control more than one land from anywhere, the concept of IoT becomes in perfect tandem with the PA applications.

In^30^, an IoT-based mobile application called LandPKS was introduced as an estimator to land potential and resilience by combining user inputs including land geographical location and his answer to specific questions concerning his land with cloud-based knowledge and similar potential information to help in selecting sustainable land management practices against the climate change effects. The system is point-based estimator, relays on simple user inputs and only on relatively static soil properties, such as soil texture and depth.

A monitoring tool called PETEFA is proposed in^31^, it exploits the data received from a meteorological station sensors, weather station sensors, and images from satellites such as LandSat 8, Spot 6 and 7, WorldView 2, PERUSAT-1 and from UAVs, to provide three types of information and spatial data for the yellow corn crops (Zea Mays) for the sake of the National Institute of Agrarian Innovation (INIA): the crop temperature and health status throughout its lifecycle, the soil state, and information on crop evapotranspiration with different Privileges for INIA and the farmers.

The work in^32^ proposes for irrigation control. It includes a web application visualizes the data comes from the field, allows for manipulating the irrigation conditions, analyzing the data to forecast the future water need using data mining. The control is automatically driven by the field data or manually through a mobile application.

An IoT-based decision support system was proposed in^33^ for mitigating the potato late blight disease and support its management. The real-time data received from the field sensors is utilized to compute the per day average temperature and number of successive hours where the humidity exceeds 90%, then these values are input to an implemented disease forecast model which computes a blight units parameter and test some conditions to send an SMS warning to the farmer when he should begin the treatment against the disease. The dashboard of the system also displays the deployed nodes in their real locations and some information about them with their real-time sensed values.

Some of the systems use a ready-to-use IoT platform as a basis for their development, some others are built from scratch; each one of these approaches has its pros and cons. While using IoT platform reduces time to market and may facilitate the IoT application development, in addition to the security issue, selecting the most suitable platform for the solution at hand is a complex task may result in inappropriate choice especially that the platform may be hard to be learnt and debugged for simple errors. In the presented solution, it is preferred to have full control and customization over its whole design, debug, update, and maintenance.

A lot of the commercial precision agriculture software programs have addressed the spatial variability analysis using the site-specific MZ approach, some of them are specialized software and some others represent integrated farm management solutions, such as the famous Trimble^34^, AgStudio^35^, AgroSense^36^, and Topcon IoT Farm Management Information System^37^. These programs depend on manually drawn zones or importing existing zones, also they enter the field data as files into the program, and may be compatible with certain type of hardware sensing devices.

Some software tools for land assessment were proposed for land use planning such as Automated Land Evaluation System (ALES)^38^ and Land Suitability Evaluation (LSE)^39^. The LSE executes on raster data not on text data, defines the land suitability classes by multi-criteria analysis and directly provides the analysis output through a map; it is flexible, supports defining and changing the values of the input variables. However, the data is entered and uploaded to these tools manually by the users, and accordingly is updated manually.

**Figure 1:**
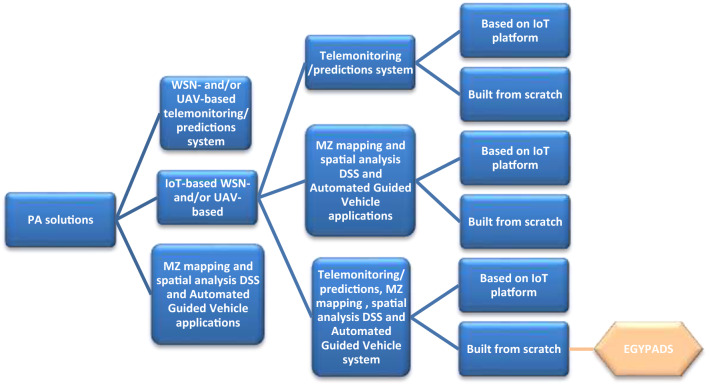
Hypothetical classification of PA solutions and identification of EGYPADS categorization.

From the above, we can find that the IoT should be a key component in a PA system, and this is realized in our PA software system. The developed system is an IoT use case in the field of agriculture provides software solution for land suitability assessment, but it is concerned with temporal and spatial analysis of soil, climate and plant data. It was designed to retain some features and functions of existing solution, improve, and add to them, while addressing their flaws; this is such as different users with different privileges, real-time update and detections, flexibility in data entry methods, suitability to more than one small to large scale land with any geometric shape, extendibility, warnings and possibility of remote control, and more. A classification represents the above mentioned PA solutions variations is depicted in Fig. 1. To the best of our knowledge, the class of EGYPADS and generally the classes of IoT-based MZ mapping and spatial analysis DSS have no outstanding agriculture-dedicated solutions examples implemented and tested^40–43^.

The features of EGYPADS are compared with a sample of PA software applications characterized by offering spatial analysis and MZs delineation services in Table 1. From this comparison, it could be noticed that LSA is not supported by the other applications or its support lacks the IoT advantages, they rely on global data sources and accordingly operate at a regional scale with the denial of near real-time and high temporal-spatial resolution advantages. Most of the applications, including EGYPADS, take care of the user convenience regarding the installation and usage of the application, therefore they replaced desktop application development with a mobile- or web-based application. EGYPADS is unique in some features such as centralized remote management of farms through different roles and authorities and the 3D virtual coordinates spatial analysis for small farms when GPS is not available.

**Table 1:** Comparison between EGYPADS and MZs delineation and spatial analysis software applications.

	EGYPADS	^44^	^45^	^46^	^47^	^48^	^49^	^50^
IoT-enabled	✓	✖	✖	✓	✖	✓	✖	✖
Desktop/Web/mobile app interfaces support	✖/✓/✖	✖/✓/✖	✓/✖/✖	✖/✓/✖	✓/✖/✖	✖/✓/✖	✖/✓/✖	✖/✓/✖
LSA support	✓	✖	✖	✖	✖	✖	✓	✖
Import /export data and MZ maps	✓/✓	✓/✓	✓/✓	✓/✓	✓/✓	✖/✖	✓/✓	✓/✓
Flexibility in formulating events	✓	✖	✖	✖	✖	✓	✖	✖
Different types of alerts	✓	✖	✖	✖	✖	✓	✖	✖
Remote control possibility	✓	✖	✖	✓	✖	✖	✖	✖
Supporting more than one land at a time	✓	✖	✖	✓	✖	✖	✓	✓
Supporting any land shape and area	✓	✓	✓	✓	✓	✓	✖	✓
Supporting more than 100 node/land or sample/land	✓	✓	✓	✓	✓	✓	✖	✓
Supporting more than one crop	✖	✓	✓	✓	✓	✓	✓	✓
Heterogeneity and large number of parameters	✓	✓	✓	✖	✓	✓	✖	✓
Historical data visualization and analysis	✓	✓	✓	✓	✓	✓	✓	✓
Virtual coordinates 3D spatial analysis	✓	✖	✖	✖	✖	✖	✖	✖
Real-time monitoring of the field nodes status	✓	✖	✖	✓	✖	✖	✖	✖
Different users with different authorities and privileges	✓	✖	✖	✖	✖	✖	✖	✖
User-friendly or ease of use	✓	✓	✓	✓	✓	✓	✓	✓
Extensibility to integrate the latest research development	✓	✖	✓	✓	✖	✓	✓	✓
Accuracy measures								
Spatial information quality	✓	✓	✓	✓	✓	✓	✓	✖
Zoning efficiency	✓	✓	✓	✖	✓	✖	✓	✓
How current is the data	✓	✖	✖	✓	✖	✓	✖	✖
Meeting required spatial resolution	✓	✓	✓	✓	✓	✓	✖	✓

Few other programs have some of the EGYPADS features such as the flexibility in formulating complete events with logical, relational, arithmetic operations and with different types of alerts and actions regarding any land registered on the system, any deployed nodes, and any phenomena, with timing constraints. Also, few sample programs offer remote monitoring of the field sensing devices status.

The presence of some common characteristics between EGYPADS and other programs does not mean that they provide them in the same way and capabilities, for example, in EGYPADS, we can easily register/unregister any land of any area or geometry so that it takes advantage of all the provided system services, easily navigate to them, and visually compare the adjacent lands and their updates in real-time, without having to create different project for each land or thematic map.

EGYPADS is not limited to certain devices like other commercial software programs, it exposes interfaces to devices that have all or some of its supported sensing modalities. These devices can communicate with the application, if these devices use the appropriate IoT client library and settings, use the secret land code dedicated to the farm that contains them, adhere to the employed topic tree naming conventions, and use the application payload structure. The LSA function of the EGYPADS current version is developed for the potato crop, therefore it needs to be enhanced with a support for different crops; also forecasts is a service offered by some other programs but missed in EGYPADS.

Table 1 also mentions the MZs accuracy measures of the systems in terms of spatial information quality, zoning efficiency, freshness of data, and spatial resolution. The spatial data quality is determined by the use of spatial dataset filtering to eliminate noisy data and outliers. Some software applications include filtering as a function precedes data interpolation; some of them implements the global outliers elimination without consideration of local outliers. The systems that depend on acquiring data from infield sensing devices through periodic sensing application, such as EGYPADS, can implement filtering at the field data sources, and may be through different levels of aggregation or forwarding.

The location accuracy, defined as the closeness of the information on maps to the values in the real world, is also an important determinant of the spatial data quality. The potential gross error caused by the human factor in taking and recording the geospatial readings using handheld devices threatens the location accuracy of systems that relay on this for dataset collection; whereas calibrated infield sensor nodes in IoT-enabled systems, such as EGYPADS, is free from this error, and an estimated redundancy of sensor nodes can compensate for any environmental or instrumental errors and increase data reliability. A system in Table 1 is marked as having good spatial information quality if it incorporates filtering procedures or location accuracy ingredients.

The zoning efficiency or spatial analysis efficiency lies in the interpolation process and the aggregation of multiple layers of information to produce the management zones that incorporate experts knowledge. The spatial interpolation of data before MZ delineation is a required step for improving isolines smoothness and for variance reduction. The most commonly used methods for interpolation are the deterministic Inverse Distance Weighting (IDW) and the geostatistical kriging methods. Kriging is more suitable for large datasets, but causes problems with the presence of outliers. IDW is more suitable and gives better accuracy for homogenously distributed small digitized datasets, as in EGYPADS. The system is marked in Table 1 as producing efficient zones when it employs interpolation and layers aggregation procedures. Freshness of data as well as the temporal resolution is intuitively better in IoT-enabled systems. In the same systems, the required level of spatial resolution can be controlled by adjusting the number and distribution of active sensor nodes.

## Materials and methodology

The field management is a complex process related to several factors such as soil conditions, crop types, climate conditions, and water quality. The current case study is related specifically to soil suitability evaluation for potato crop. However, the study generally addresses the spatial analysis of the agricultural phenomena, such as the chlorophyll content index (CCI) and the atmospheric temperature, as well as the generation of their corresponding management zones maps. This section describes the study area, EGYPADS system architecture, and its spatial analysis and land suitability evaluation models that are used to delineate the management zones.

### The description of study area

**Figure 2:**
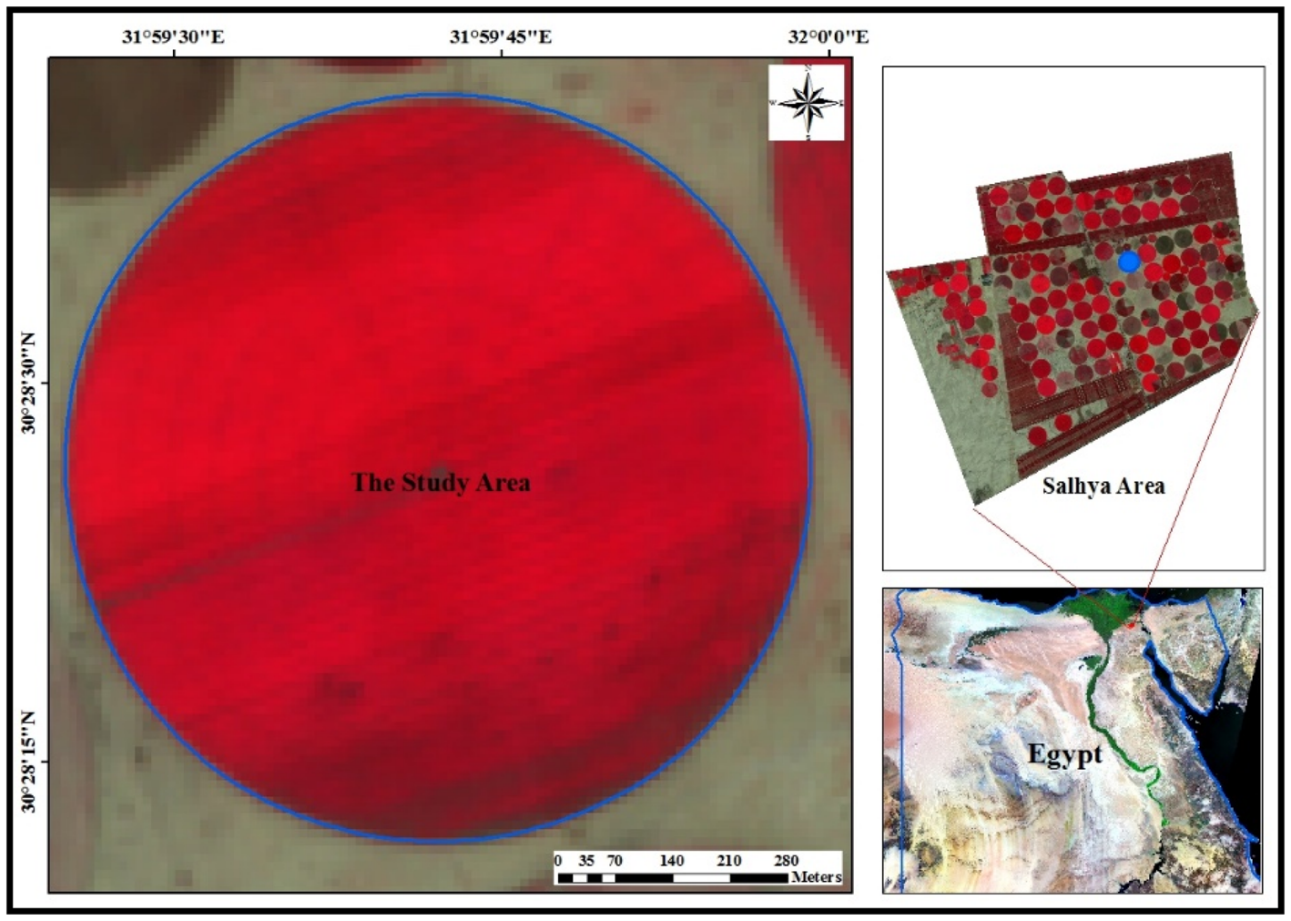
Location of the studied area.

The study area is located among the soil of Sixths of October Company for Agricultural Projects (SOAP) which is located in El-Salhia Area, south west of Ismailia city and to the East south of El-Kassaseen city. It is bounded by 30° 28′ 11.93'' and 30° 28′ 41.63'' latitudes and 31° 59′ 25.31'' and 31° 59′ 59.45'' longitudes as shown in Fig. 2.

### Field trips and laboratory analysis

**Figure 3:**
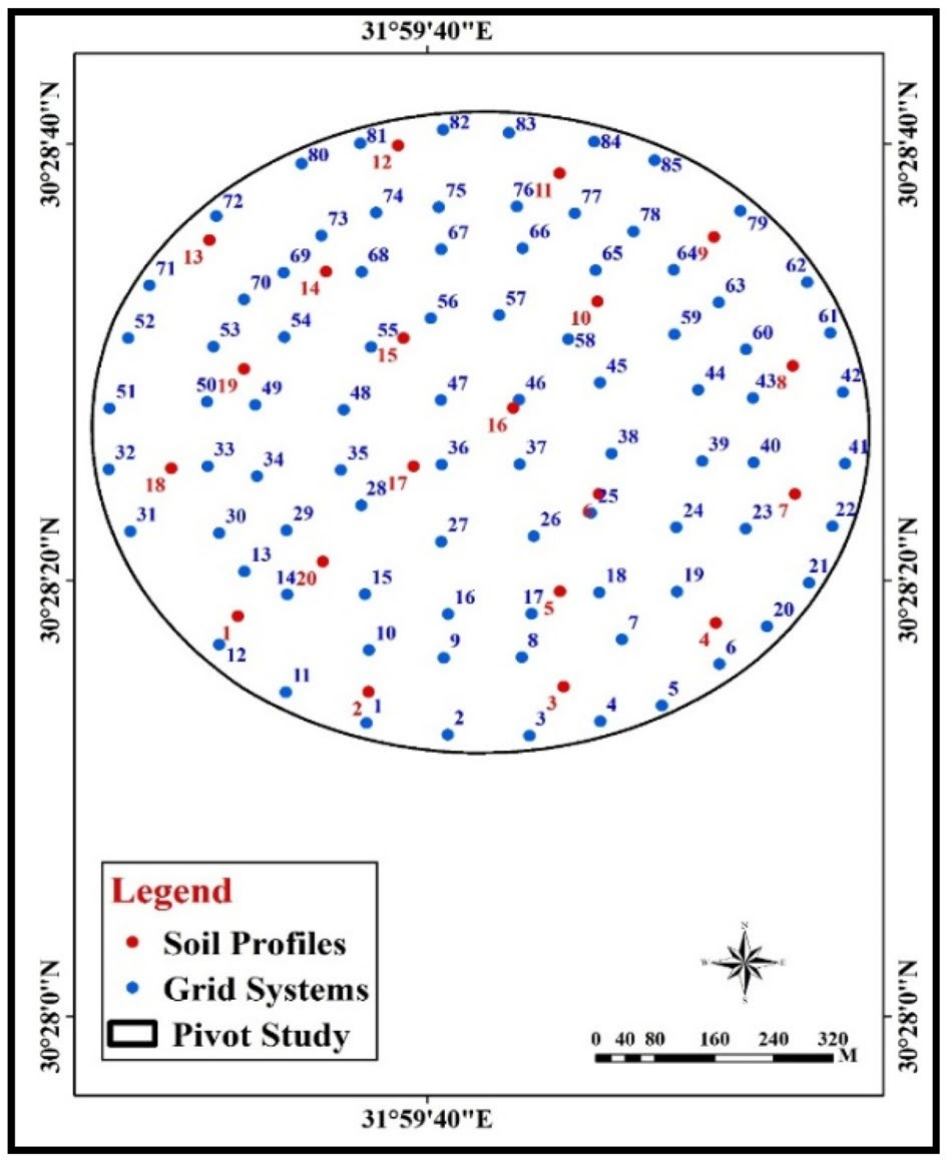
Soil profiles and grid systems of the study area.

A number of five field trips include on-site field measurements were conducted for different crop growth stages: during five stages after 30, 45, 60, 80, and 90 days from planting. The field trips post-activities include laboratory analysis for collected soil profiles, and spatial analysis for the study parameters. Twenty soil profile were carried out as they represent the variation of soil characteristics of the selected farm, the study area was divided into equal cells based on grid system decided by the research team. Eighty-five sites were chosen as the main dataset for the modeling process during different growing stages of potato crop. These sites represent all possible variations in soil conditions and agricultural practices which may occur within the experimental fields. The grid system and the location of the check points are explained in Fig. 3.

A total of 155 soil samples were collected from soil profiles and surface samples. In addition to 425 plant samples were collected during growing stages of potato crop (5 stage × 85 samples) and analyzed for physical and chemical characteristics using the standard analytical methods as indicated by^51^**.**

### The proposed overall system architecture, requirements, and functionality

**Figure 4:**
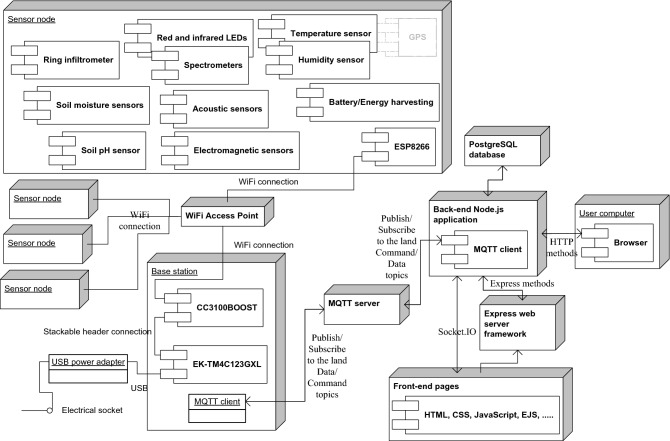
The system deployment diagram.

The EGYPADS represents a web application integrated with the IoT technology to receive the data from infield devices directly. The technical description of the system is analyzed into two main parts: the front-end and the back-end. Figure 4 shows the application main modules and its relation to the other system components.

The back-end of the system was developed using Node.js server environment. As shown in Fig. 4, The JavaScript back-end application receives the field data stores it in a database and forwards the required data to the front-end through socket communication to be displayed in near real-time. The Express server^52^ renders the front-end webpages and through which the application receives the POST data sent from the front-end to the back-end, then it appropriately deals with it.

The system uses:

- *PostgreSQL*^53^ a general purpose and object-relational database management system; the most advanced open source database system.
- *Message Queuing Telemetry Transport (MQTT)*^54^ a lightweight publish/subscribe messaging transport protocol for machine-to-machine or IoT connectivity protocol on top of TCP/IP.
- *JavaScript Object Notation (JSON)*^55^ a lightweight data-interchange format
- Front-end development languages and tools such as the HyperText Markup Language (HTML), Cascading Style Sheets (CSS), Bootstrap CSS framework^56^, JavaScript, the Embedded JavaScript templating (EJS)^57^ which is a simple templating language allows for generating HTML markup with plain JavaScript.
- *Mapbox* a mapping platform.

The following are required for the system setup:

Node.js, the npm package manager (included in Node*.*js installation), the PostgreSQL relational database management system, dedicated server connected to the internet with real public IP address to run the application.

EGYPADS is a software system prepared to be a part of the large IoT system, therefore it exposes interfaces for field devices communication. The MQTT client of the back-end subscribes to the topics to which the registered lands publish their data, while the MQTT clients of the registered lands' base stations subscribe to the topics to which the back-end publishes commands to these lands' devices. Any land registered to the system is given a secret code shared only between the land base station and the back-end application. This land code is used to authenticate it and identify its data at the back-end, and it is embedded in the topic tree naming convention employed in each EGYPADS system instance. As shown in Fig. 4, the things IoT tier is composed of a base station connected wirelessly to sensor nodes deployed in the land. In the validation test in this paper, the base station is the Texas Instruments CC3100 WiFi transceiver board connected to the ARM Cortex-M4F based MCU TM4C123G LaunchPad evaluation kit (EK-TM4C123GXL).

In the current version of EGYPADS, the system accepts values of two climate parameters: temperature and relative humidity, one plant parameter which is the CCI, and the seventeen soil parameters. The seventeen soil parameters are used to estimate the soil quality indices and assess its suitability: phosphorous (P), organic matter (OM), nitrogen (N), potassium (K), zinc (Zn), drainage (R), texture (T), depth (D), topography (F), surface stoniness (Y), hard pan (HP), hydraulic conductivity (G), water holding capacity (WHC), salinity (S), exchangeable sodium percentage (ESP), CaCO3, and pH (PH). The values of some of the soil parameters are only deduced through laboratory analysis of soil samples, therefore the system allows for entering the values of the laboratory parameter of each land via an excel sheet of an application accepted format. A sensor node may contain all the mentioned sensing modalities, or the land may contain different types of sensor nodes according to sensing modalities incorporated into each type. It is also worth noting that it is not necessary to include all the scores of three soil quality indices, but some soil parameters can be neglected and accordingly their scores omitted from the index equation with modifying the equation exponent (omitting a parameter score from the equation entails reducing the denominator of the exponent by one).

EGYPADS expects values for all parameters in the message payload: the sensed parameters' values, otherwise NULL, however the value is retrieved from the database if it is a laboratory parameter. The payload JSON content contains the identification of a land and a node together with the node real coordinates and sensed data.

**Figure 5:**
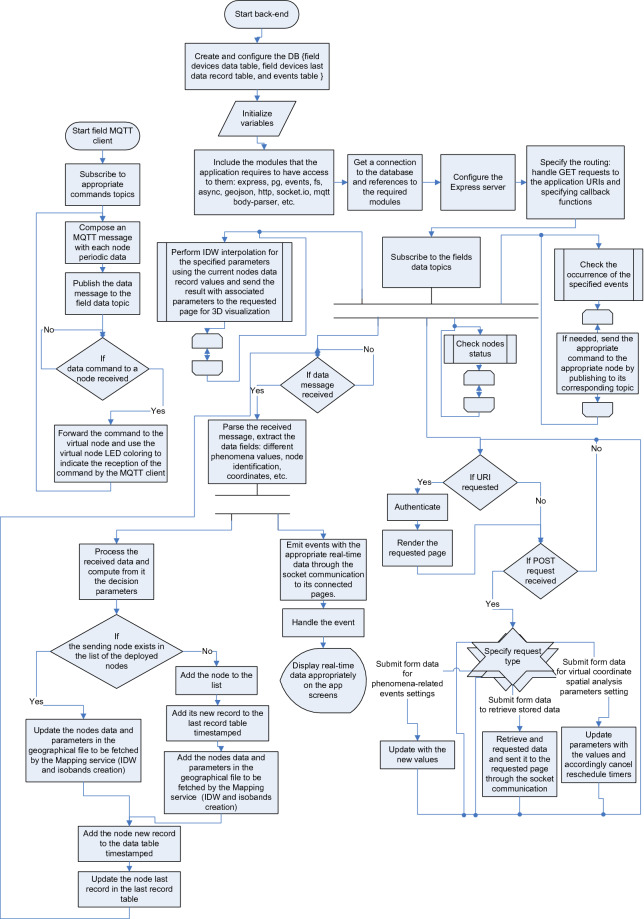
Heuristic diagram of the system main operations.

The sensor node may contain actuators to actuate field equipments such as sprinklers. The sensor node may not contain GPS and thus uses the Virtual Coordinates Mapping Service as will be illustrated later. The soil moisture sensors can be used to measure the WHC, acoustic sensors can be used to measure T and D**,** ring infiltrometer can be used to measure hydraulic conductivity G, electromagnetic sensors measure various soil properties that, such as T, R, OM, S. The red and infrared LEDs together with the two corresponding spectrometers can be used to measure the CCI. The back-end JavaScript application manages all the system operations and performs behind-the-scenes functionality that offers EGYPADS services to the users and allows them to interact with the system. An overview of EGYPADS functionality is depicted in Fig. 5 flowchart.

EGYPADS back-end application starts with the required configurations, initializations, access to modules, URI Routing settings, subscription to the MQTT topics of fields data; then, it starts its parallel operations. It performs the periodic operations of checking nodes status, checking for the occurrence of specified events, and performing calculations for the 3D virtual coordinates data charts. In flowchart of Fig. 5, the "Loop limit" shape and the associated inverted one indicate the start and end of a loop containing the procedures of an operation, respectively. The back-end application always listens for incoming requests to display HTTP URLs, or incoming data through the POST method to handle it appropriately whether it is data for updating the virtual coordinates chart settings, a request to retrieve stored data, a syntax of a specific event to be registered, etc.

Likewise, the back-end application always listens for incoming data messages from the fields. Once a data message is received, it is parsed, the real-time values of parameters are issued to be displayed in the system screens, the sending node record is added to the database or updated if already exists, and the geographical file data is updated to be ready to be fetched by the mapping services.

While that the server tier of the system starts and performs its operations, as shown in Fig. 5, the MQTT clients of the fields also start and perform their operation by subscribing to command topics, publishing periodically to fields data topics, and listening for incoming command messages.

### Land management zones delineation using EGYPADS

The land management zones were delineated based on land suitability criteria of the potato crop according to^51^. Thirteen parameter have been used in this work to study land suitability for potato. These parameters are OM, N, P, K, Zn, R, T, D, G, S, ESP, CaCO_3_ and PH. Three thematic indicators were used in assessing land suitability: soil fertility, chemical and physical quality indices according to the following equations^58^. Equation (1) was used to calculate land suitability spatial model:

1 $$\mathrm{LS}={(\mathrm{FQI }\times \mathrm{ CQI }\times \mathrm{ PQI})}^{1/3}$$

where LS is land suitability, FQI is fertility quality index, CQI is chemical properties quality index and PQI is physical properties quality index. The physical properties quality index was calculated using Eq. (2):

2 $${\mathrm{PQI }= ({\mathrm{S}}_{\mathrm{R}} \times {\mathrm{S}}_{\mathrm{T}}\times {\mathrm{S}}_{\mathrm{D}} \times {\mathrm{S}}_{\mathrm{G}})}^{1/4}$$

where S_R_ is the score of drainage, S_T_ is the score of Texture, S_D_ is the score of soil depth, and S_G_ the score of Bulk density. The chemical quality index was calculated using Eq. (3):

3 $${\mathrm{CQI }= ({\mathrm{S}}_{\mathrm{S}} \times {\mathrm{S}}_{\mathrm{E}}\times {\mathrm{S}}_{\mathrm{K}} \times {\mathrm{S}}_{\mathrm{H}})}^{1/4}$$

where S_S_ is the score of soil salinity, S_E_ is the score of ESP, S_K_ is the score of CaCO_3_ content and S_H_ is the score of soil pH. The fertility quality index was calculated using Eq. (4):

4 $${\mathrm{FQI }= ({\mathrm{S}}_{\mathrm{N}} \times {\mathrm{S}}_{\mathrm{P}}\times {\mathrm{S}}_{\mathrm{K}} \times {\mathrm{S}}_{\mathrm{zn}}\times {\mathrm{S}}_{\mathrm{OM}})}^{1/5}$$

where S_N_ is the score of available nitrogen, S_P_ is the score of available phosphorous, S_K_ is the score of available potassium, S_zn_ is the score of available zinc, S_OM_ is the score of organic matter content and Sc/n is the score of ratios of carbon to nitrogen.

### Mapping spatial analysis of soil characteristics

Data was gathered, entered, stored, manipulated, analyzed, and output by a decision support system program. The locations of soil profiles were digitized in order to know the geographic coordinates and the weighted average value for each parameter belonging to each soil profile (soil depth, saturation percent (SP), pH, EC, OM, CaCO_3_, Gypsum, CEC, sand, silt, clay and texture) and to carry out interpolation for soil properties using the program. The weight values of selected parameters calculated in Analytic Hierarchy Process and designated scores for physical and chemical quality assessment as following in Tables 2 and 3. The same calculations of weights and scores for soil parameters, evaluation criteria, quality classes were employed in EGYPADS.

**Table 2:** Weights of the criteria and scores soil quality classes of the study area^58^.

Soil parameters	Quality class	Score
FQI	High quality	> 0.9
	Moderate quality	0.7–0.9
	low quality	0.5–0.7
	Very low quality	< 0.5
CQI	High quality	> 0.9
	Moderate quality	0.7–0.9
	low quality	0.5–0.7
	Very low quality	< 0.5
PQI	High quality	> 0.75
	Moderate quality	0.75–0.50
	low quality	0.50–0.25
	Very low quality	< 0.25

**Table 3:** LS classes of the study area^58^.

Suitability	Suitability class	Score
Highly suitable	S1	> 0.8
Moderately suitable	S2	0.8–0.5
Marginally suitable	S3	< 0.5

### Preparation for implementation and validation

**Figure 6:**
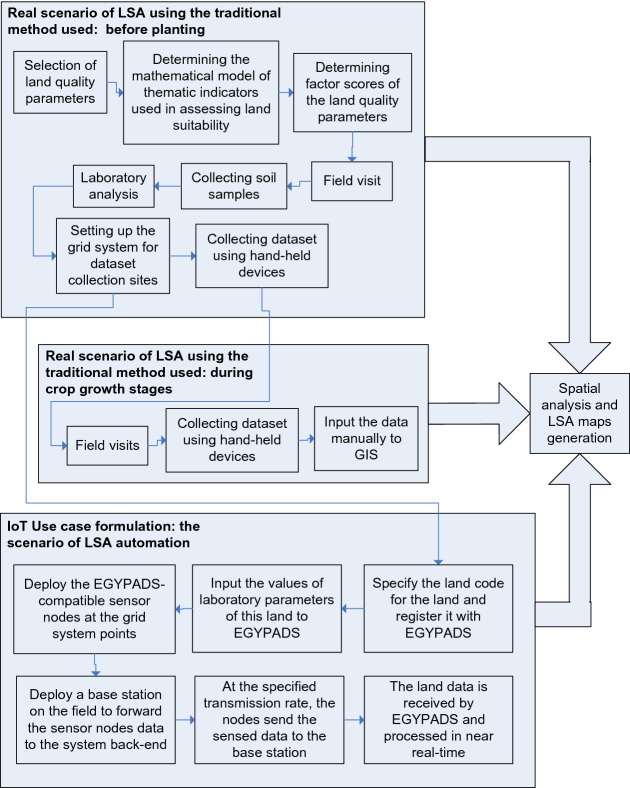
Functional relationship of EGYPADS and the traditional method.

The system implementation was preceded by using the current traditional method of performing LSA and spatial analysis, including recursive field trips, on-site data collection, and utilizing the desktop programs used to draw the analysis maps. Generating and updating these maps in real-time from the data comes directly from the field were taken as the main objectives of the proposed system. A schematic represents the main activities of using both EGYPADS and the traditional method and a relation between them is depicted in Fig. 6. The field trips were important to: know the specification of the output maps and the methods used to generate them, put the design and requirements of the whole system design, and practically understand the real benefit of the system. In addition, a field trip is essential to determine the values of the laboratory parameters.

**Figure 7:**
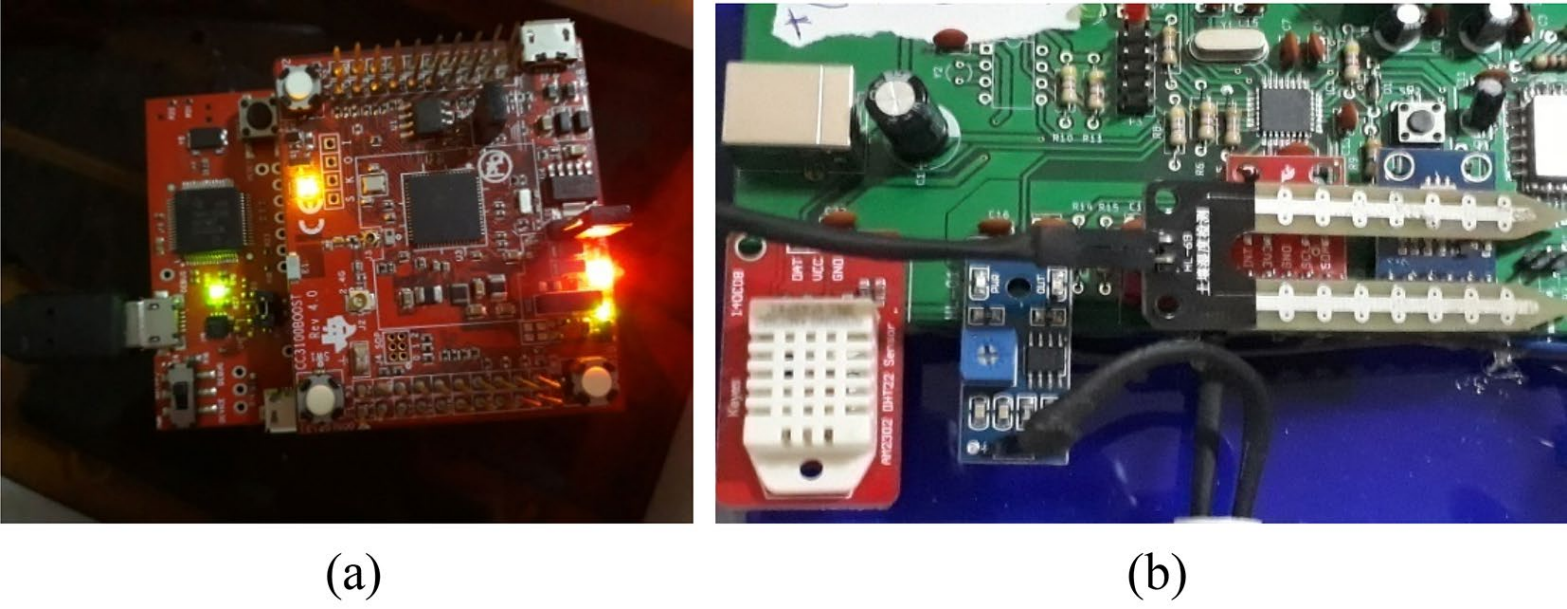
The hardware used in the validation test, (**a**) the base station, (**b**) a sensor node with a temperature and soil moisture sensors.

**Figure 8:**
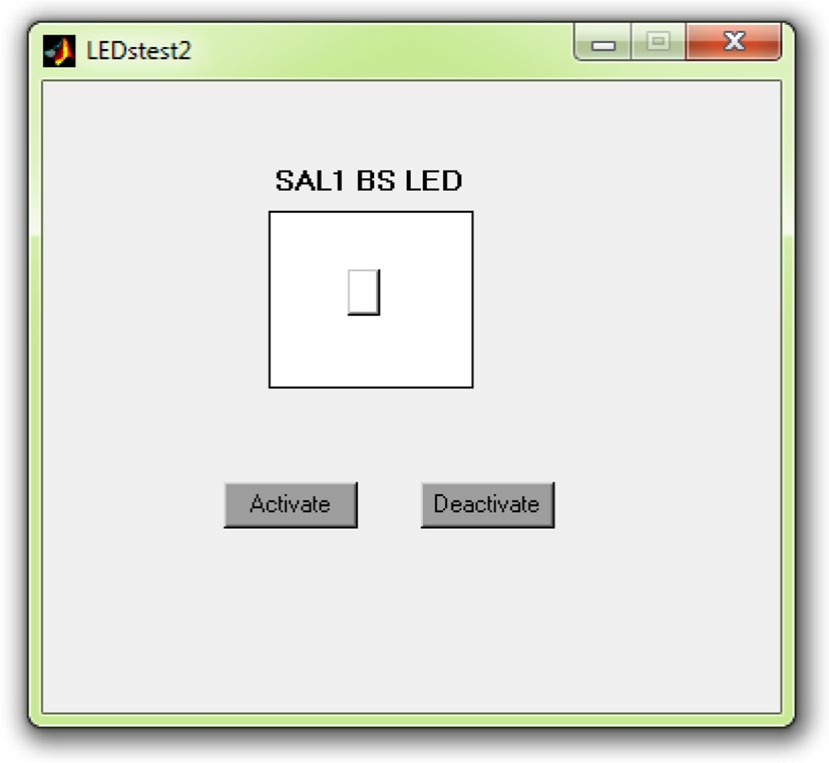
Virtual node LED indicator.

To test and validate the EGYPADS system functioning, the IoT connectivity was implemented using a CC3100-TM4C123G base station connected in a star topology to three sensor nodes contain DHT22 temperature sensors and LM393 soil moisture sensors (Fig. 7). At the same time, in order to include different lands in the validation, the IoT connectivity was also emulated by employing a real MQTT connection, can run on different computers (work as the field devices), and sends its packets to the application over the Internet; while the functionality of these virtual field devices is simulated by MATLAB software coding with programs represent a light-emitting diode (LED) component on the devices (shown in Fig. 8). This LED coloring is used as an indication to the reception of a remote command from the application to this device.

Each of these virtual devices can work as an IoT-enabled node deployed on the field sends its data directly to the system through the Internet or an IoT-enabled base station sends the data of the field devices on behalf of them, each node data in a separate packet. The payload of the published data packet is a JSON object contains the name/value pairs the system is familiar with which contains the land code, the device identification, the GPS latitude and longitude coordinates of the device, and the values of the sensed phenomena. At the transmission time, each virtual device picks a random value of each sensed phenomenon within its acceptable range, and sets the value of the unsensed phenomenon to null.

The validation tests includes validating the system IoT connection, making sure that the system functions really work focusing on its two most important functions: the Mapping service and Events and Actions service, and emphasizing that these functions produce their required output.

**Figure 9:**
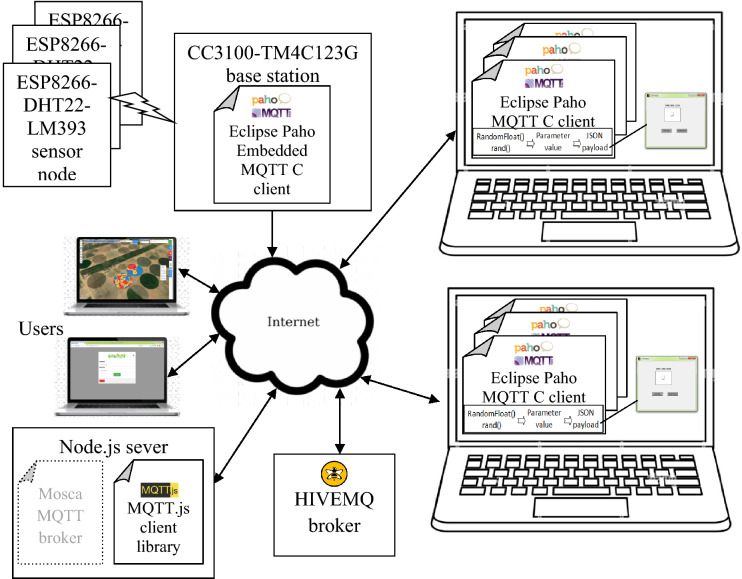
System validation setup block diagram.

The previously mentioned setup for validation is illustrated in more detail in Fig. 9. The MQTT client is implemented in the IoT devices used in the testing whether it is the embedded base station (Eclipse Paho Embedded MQTT C client) or the laptops acting as virtual base stations (Eclipse Paho MQTT C client). Two laptops were used; each laptop runs more than one virtual base station program send periodically messages of nodes data and assign random values for their parameters. The MQTT.js client library is used in Node.js back-end application.The Mosca MQTT broker^59^ is used to implement the MQTT server in a localhost machine, but to use real Internet connections between the back-end and the IoT devices, a public MQTT Server: mqtt://broker.hivemq.com is employed for the purpose of testing and validation.

## Results

Based on the analysis of the field trips data, the management zone maps were produced and the spatial distribution analysis was conducted. The analysis results can be used to decide the appropriate land use and the appropriate site-specific land management. Three zones were recognized of the study area as follows:

*Zone (I)* They represent highly suitable areas. It has very good and suitable for the potato crop. This zone is characterized by a flat area, light texture and deep soil. Their contents of salinity, carbonate, and gypsum are very low with slopes < 1%. This zone is covering about 5% of the total area. These results agree with some researches on the same area^60^

*Zone (II)* The soil of this class has one slight limitation that restricts its use for the production of the potato crop. This zone is characterized by deep to moderately deep and the texture is sand, sandy loam, and loamy sand. Their contents from carbonate range between low to moderate and gypsum are very low, the organic matter range from very low to very moderate, this limitations are dominant in Elsalhia area^61,62^. This zone occupies most of the study area is covering about 65% of the total area.

*Zone (III)* They represent moderately suitable areas for some crops. It has one or more severe limitation that excludes the use of the land that require special management practices or severely restrict the range of crops such as salinity, where the electric conductivity values range between moderate-high to moderate, and content of clay is low to very low where texture for this zone range from sand to loamy sand. This zone is characterized by moderately deep. This zone is covering about 30% of the total area).

The proposed system implementation allows to generate these maps and make such analysis continuously based on the near-real-time data sent by the devices deployed in the field, in addition to the system providing other services. The following subsections show the results of the system implementation and validation.

### The system services screens

This section describes the result of the system implementation from the three different views of internet users, normal users, and administrator.

#### The internet users view

**Figure 10:**
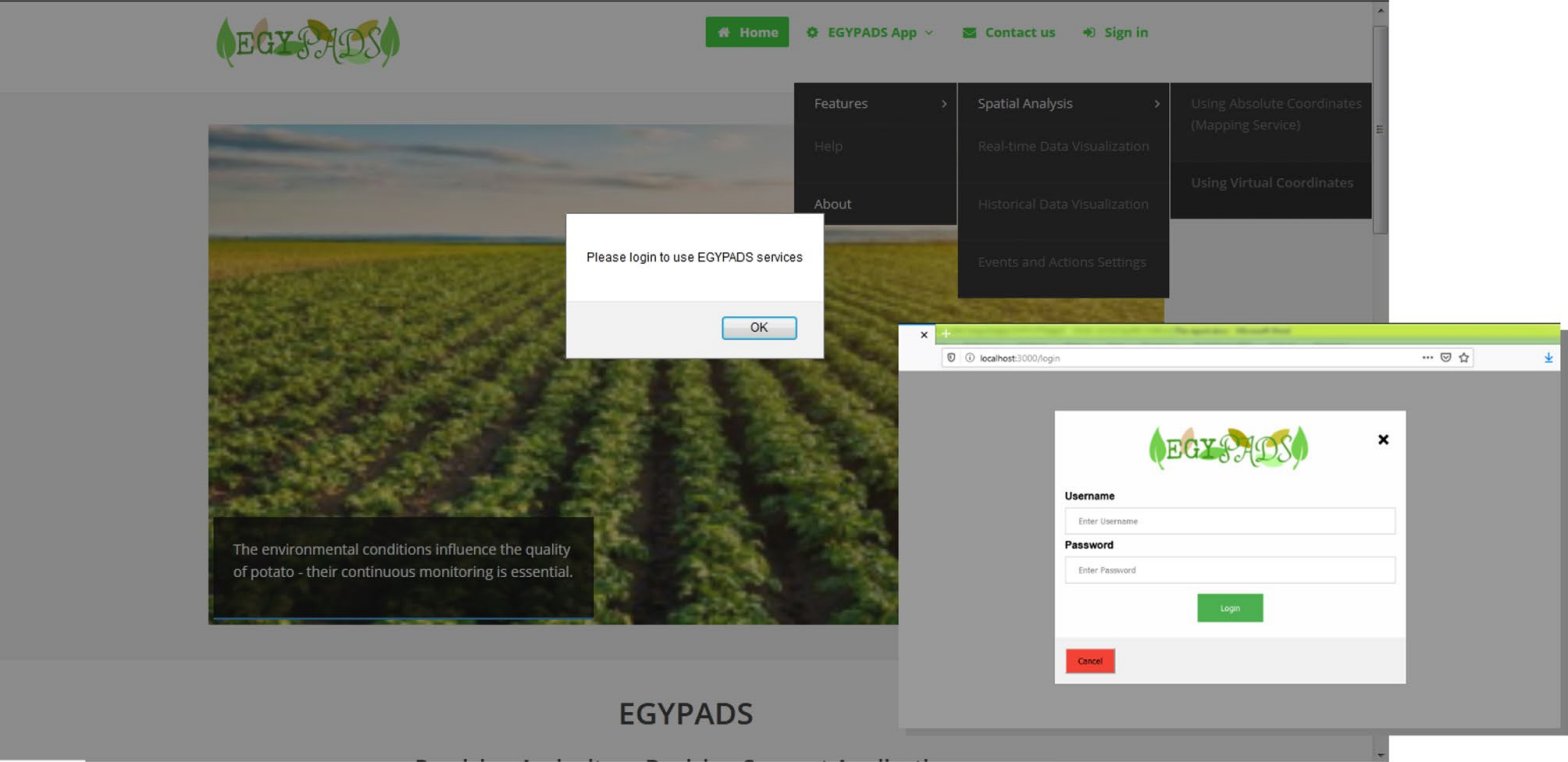
Application homepage and login page screenshots.

EGYPADS website contains a landing page available to all Internet users who can navigate through its public pages such as " About us" and "Contact us” and know about the application features and the sponsoring organizations. The application services and features reside under the "EGYPADS App" tab in the top menu. The sub-menus' links appear dimmed unclickable to the Internet user and request from him to login as Administrator or Normal user by clicking the "Sign in" tab, see Fig. 10.

#### The normal users view

After the user signed in, all the links become active to the Administrator account, but the Normal user account is unauthorized to open the "Events and Actions Settings" service pages.

**Figure 11:**
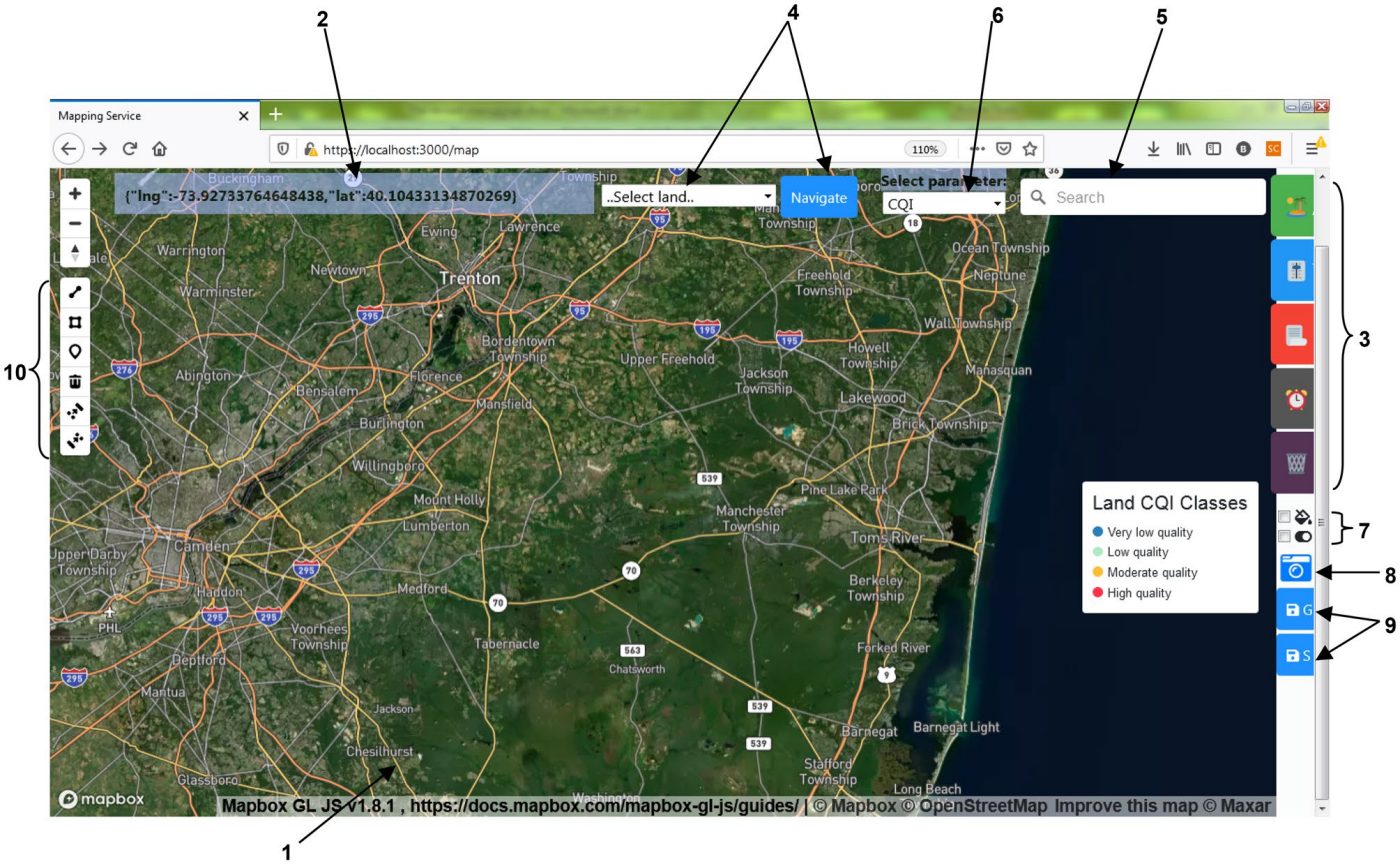
The mapping service main page.

The spatial analysis of different field phenomena and parameters can be achieved in two forms: using a geographic coordinate system through the Mapping Service or using virtual coordinates. The Mapping Service page is shown in Fig. 11. Number 1 in Fig. 11 symbolizes the real earth map. The hoverable side navigation menu, represented by Number 3, contains buttons—from top to bottom—for adding lands, adjusting the system controllable parameters values such as interpolation parameters, input all the data to be drawn from excel sheet instead of real-time field data, draw maps from historical data, and deleting land.

Number 4 and Number 5 are used for navigation. Number 4 allows the selection of a particular registered land to navigate to. Number 6 is used to select the analysis phenomena or index. Number 7 are toggle buttons to change zones opacity-level to transparent/nontransparent and to show/hide nodes positions on the map, Number 9 represents two buttons used to export maps in GeoJSON and Shapefile format. Number 10 is a set of drawing tools related to Add land button as described in the following.

**Figure 12:**
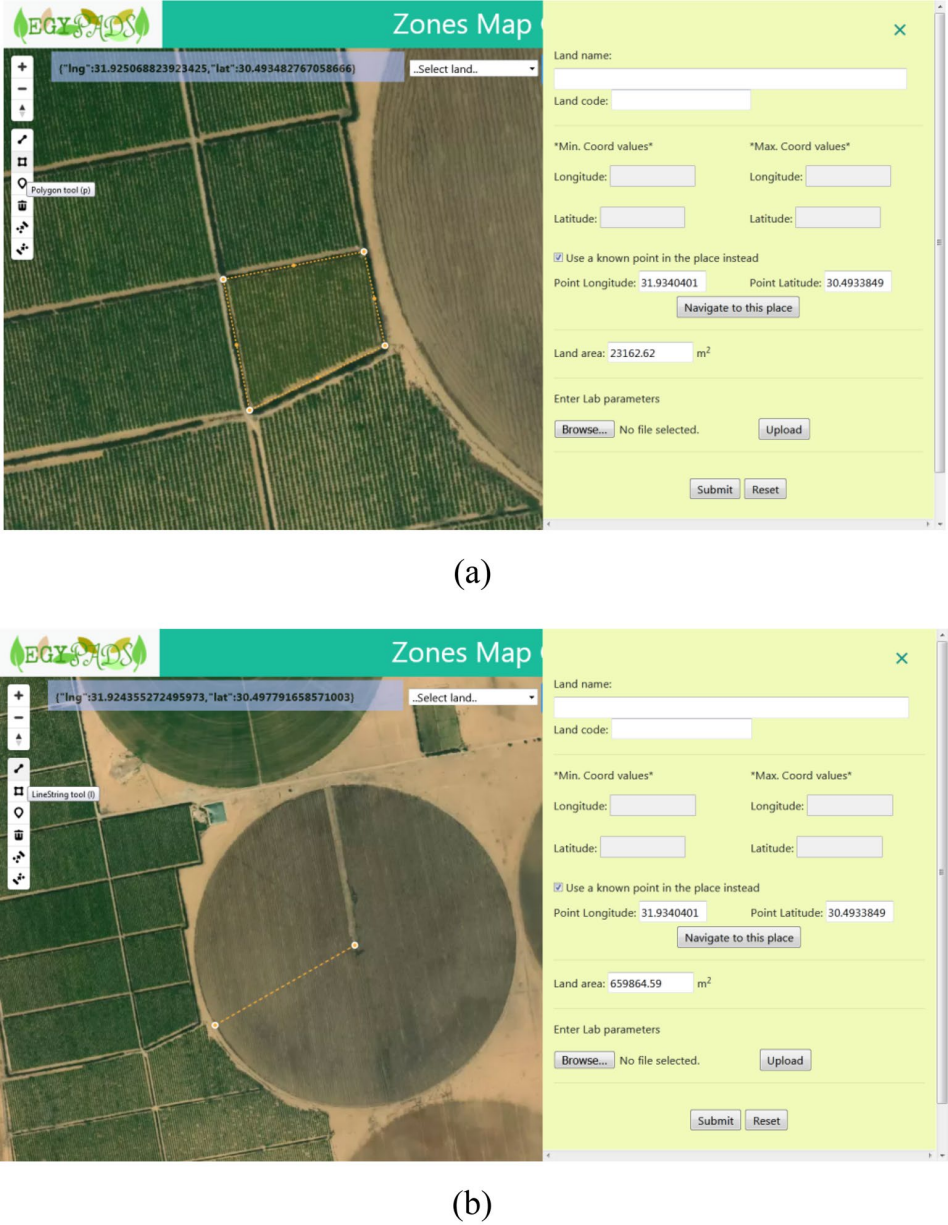
The Add land side screen, (**a**) specifying polygonal land perimeter, (**b**) specifying circular land perimeter.

By clicking the Add land button, the side screen shown in Fig. 12 appears. Through this screen, we can enter the data of the lands to be registered with the system. The land data include: a mandatory Land name which is any name selected to easily identify and discriminate lands, a mandatory Land code which should be the same as the land code included in the data messages received from the devices of this land, and the navigation information which can be the bounding minimum and maximum Longitude and Latitude coordinates or instead just the Longitude and Latitude coordinates of a certain point in the land whether known or determined by the point marker tool, and the laboratory parameter values entered by browsing to select a fixed-structure excel file. The Land area is computed automatically, this needs navigation to the land and specification of its perimeter using the drawing tools, Number 10, by moving the polygon tool on the polygonal land perimeter, Fig. 12a, or by drawing a line representing a circle radius starting from the center of a circular land using the LineString tool, Fig. 12b.

**Figure 13:**
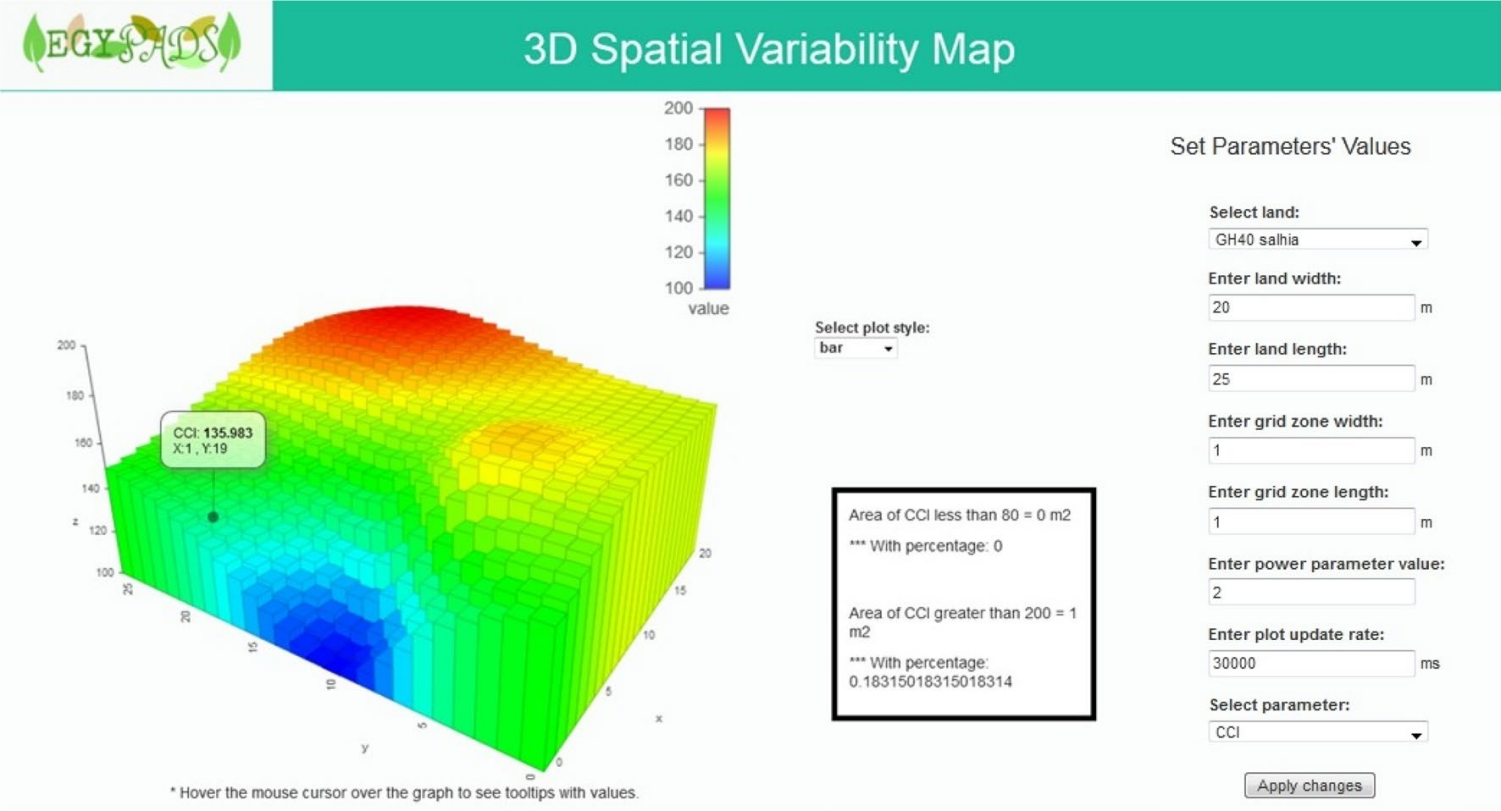
The Virtual Coordinates service page.

The spatial analysis using virtual coordinates system, where an origin point (0,0) is specified on one of the field corners and the x-axis and y-axis are determined, is useful in case of the GPS is not available and the field area is not very large and doesn't use smart equipments. This Virtual Coordinates service uses this relative coordinate to perform spatial analysis of the phenomenon or parameter using the IDW interpolation. Then the result of the analysis is visualized using surface or bar plots or other 3D plots as shown in Fig. 13.

**Figure 14:**
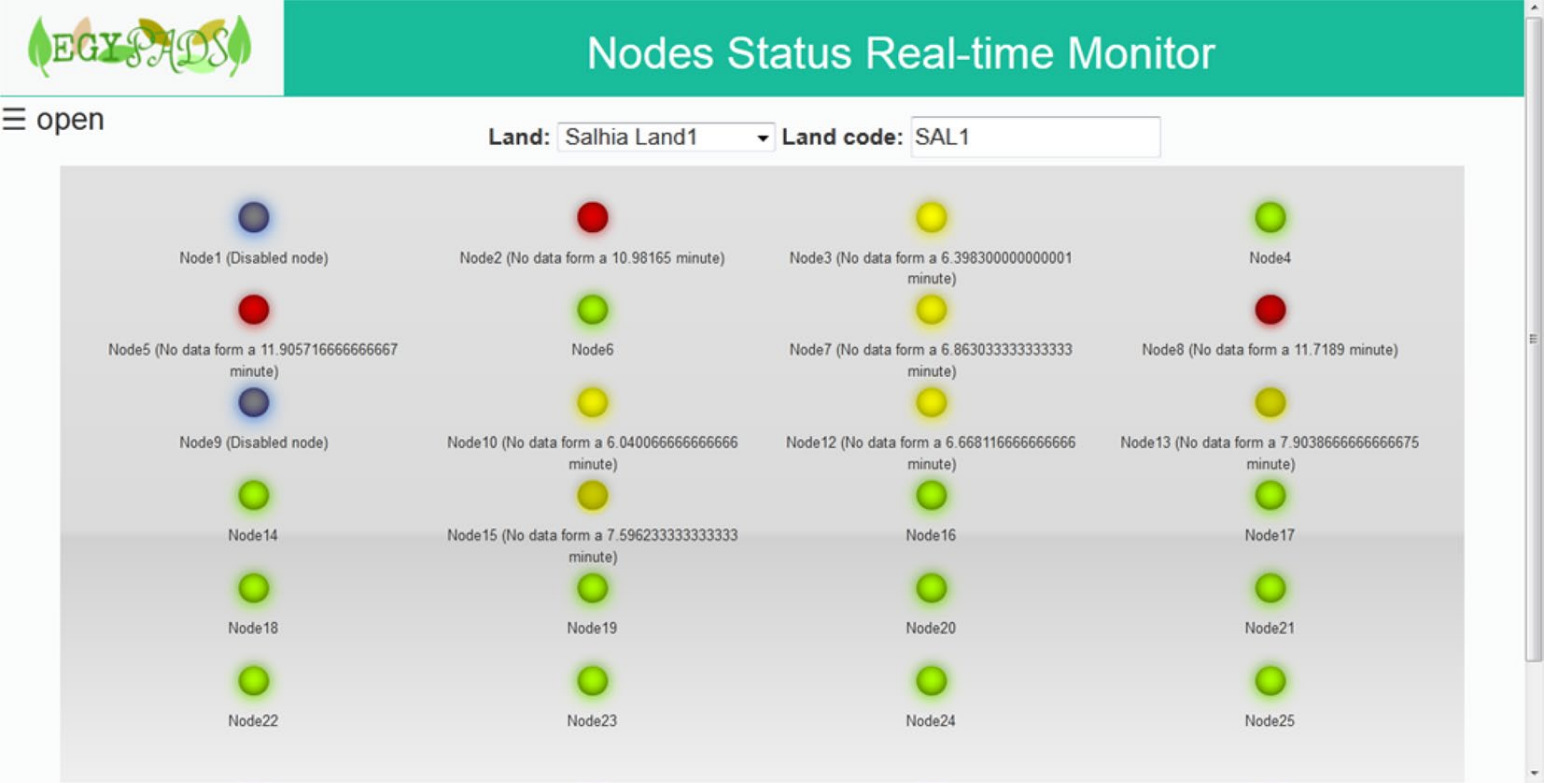
The sensor nodes' status monitoring panel.

The Real-time Data Visualization service opens on the monitoring panel of the nodes' current status. This panel is automatically populated with the nodes of the selected land as they are deployed in the field and start sending their data. The node is represented by a colored lamp; the green lamp means no severity. There are three levels of severity indicated by, in order of severity from low to high, flashing yellow lamp, flashing red lamp, dark grey lamp color which indicates a disabled node, see Fig. 14.

**Figure 15:**
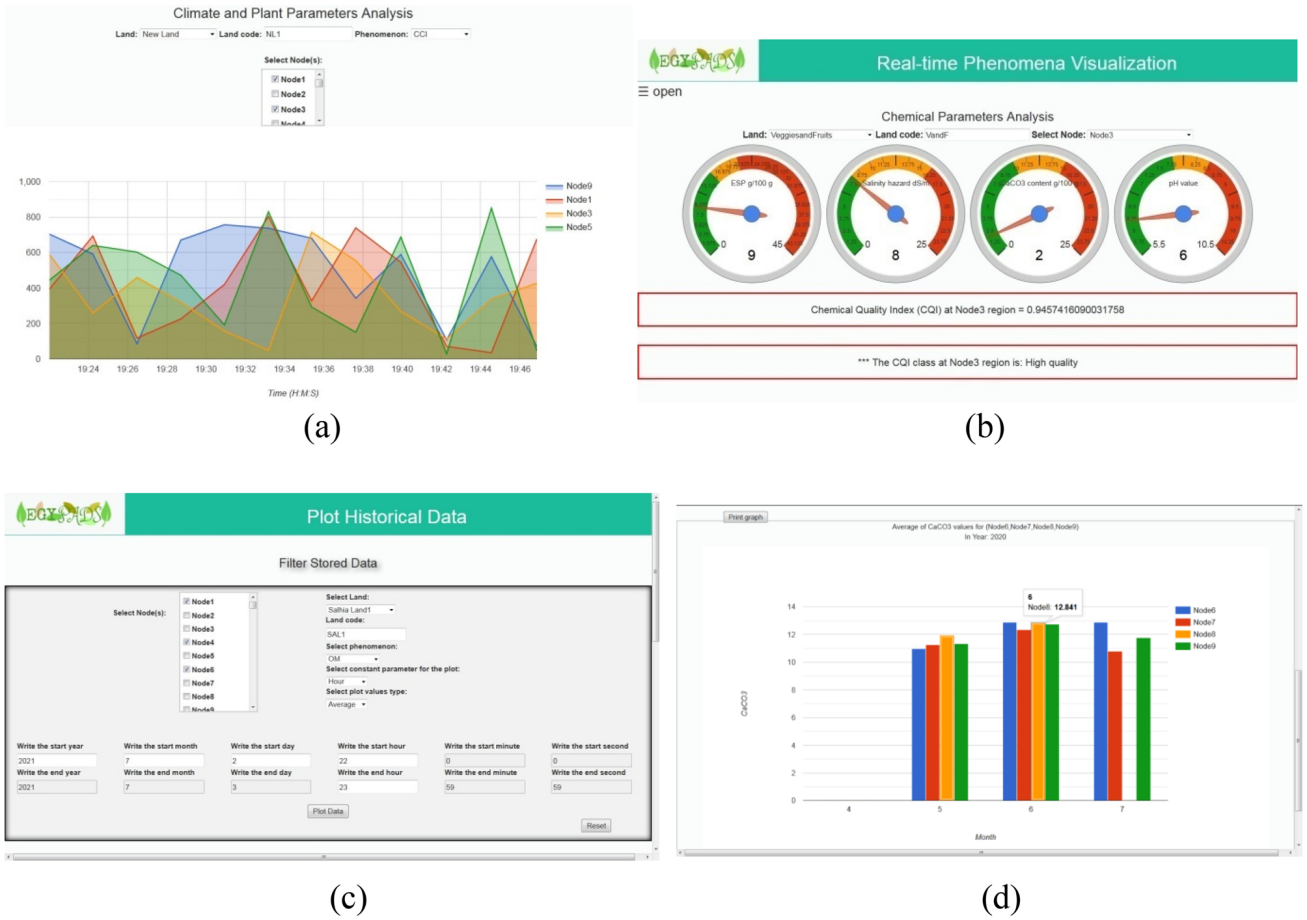
Screenshots of system services, (**a**) the climate and plant parameters real-time visualization service (**b**) the soil chemical parameters real-time visualization services, (**c**) the historical data visualization service " Filter Stored Data " part, (**d**) the historical data visualization service " Plot Area " part.

Using the top icon "open" will open the side menu to navigate through the other Real-time Visualization services. The Historical Data Visualization service aids in retrieving, plotting, analyzing, printing the data stored in the database; see Fig. 15.

#### The administrator view

The Administrator can use all the mentioned services in addition to add/delete normal users, change passwords, update some of the system parameters, and use the Events and Actions service.

**Figure 16:**
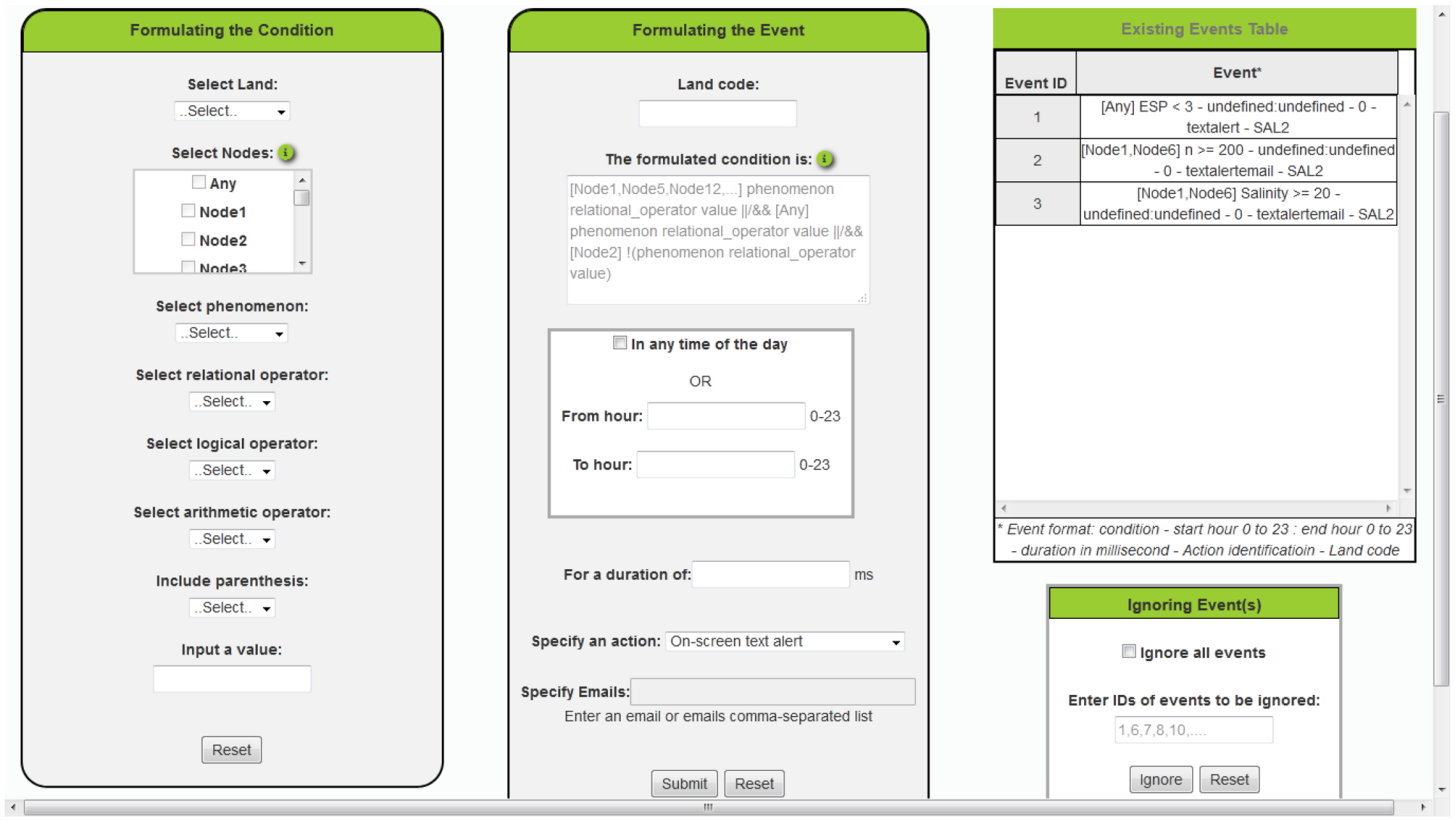
The events and actions settings service page.

This service, shown in Fig. 16, aids the administrator in the formulation of complete events with test conditions, timing constraints, and actions. The system accepts only a specified format for the condition. A list of the nodes meant by the event separated by comma between square brackets is required, or the word "Any" between the square brackets to attribute the event to any node in the land. This is followed by a relational expression consists of the name of a phenomenon as defined by the application, or a mathematical expression of phenomena, followed by relational operator and value. The previously mentioned formula can be repeated as much as the event entails separating them by the logical operator OR or AND. Also, the logical operator Not can be used to reverse the result of a relational expression.

The administrator can write the required condition directly in the placeholder "The formulated condition is" or it is better to use the facility given by the application in the first form "Formulating the Condition" which enables him to only select what he wants to include in the condition and it will be written on behalf of him in the application accepted format.

The timing constraints of the event include specification of the time of the day in which the event should be evaluated and whether it is required for it to remain true for a duration or the action is taken once it is evaluated to be true.

### Implementation validation results

This section is dedicated for checking that the implemented system meets its requirements and objectives. The scenario, steps, and results of the validation tests are illustrated in the following subsections.

#### The validation test1 scenario

Suppose a scenario incorporates two lands, in each land there is a base station sends the data of the deployed sensor and actuator nodes on behalf of them to the system server. Table 4 shows some descriptive parameters of the two lands and the test.

**Table 4:** Parameters of the validation scenario.

	The first land (The same land that was employed for the field trips)	The second land (Adjacent land)
Land name	Salhia land1	Salhia land2
Land code	SAL1	SAL2
Land area shape	Circular	Circular
Navigation information	Bounding minimum and maximum Longitude and Latitude coordinates: 30.4701 and 30.4782 Latitudes, 31.9891 and 32.001 Longitudes	Longitude and Latitude coordinates of a point: 32.004 Latitude and 30.4776 Longitude
Number of deployed nodes	85	11
Laboratory parameters	ESP, CaCO3, N, P, K, Zn, R, F, HP	ESP, CaCO3, N, P, K, Zn, R, F, HP
Transmission rate	10 s	10 s
IDW exponent	2 (the default)	2
Interpolation grid cell size	0.01 km (the default)	0.01 km

#### The validation test1 steps

The following steps and analysis are used to validate the system IoT connection including the publish and subscribe functions, ensure the work of system functions focusing on the Mapping service and Events and Actions service, and ensure the correctness of the functionality.

- *Validation of IoT connectivity and system functionality for different lands*

*Step 1* Register Salhia land1 by submitting its information with the system using the "Add land" button.

*Step 2* Register Salhia land2 by submitting its information with the system using the "Add land" button.

*Step 3* Run the Eclipse Pahoclient represents the base station of Salhia land1 from a computer.

*Step 4* Run the Eclipse Pahoclient represents the base station of Salhia land2 from another computer with different internet connection.

*Step 5* Open Events and Actions page, delete any existing event, formulate the following hypothetical events:

*For Salhia land1 and Salhia land2* if half of the Nitrogen value minus 0.02 is greater than or equal the potassium value multiplied by 1.2 and the organic matter is less than 0.5 for Node1 and Node6 and Node10, in the first half of the day for Salhia land1 and in the second half of the day for Salhia land2, send immediately command close valve to Salhia land1 base station and command open valve to Salhia land2 base station.

*For Salhia land2* if temperature value of any node happens to be smaller than 10 °C and remains the same for 1 min in any time of the day, issue on-screen text alert together with email alert message.

- *Functionality correctness validation*

*Step 6* Validate the Export format by exporting a management zone map either in GeoJSON and Shapefile formats, and open it by another program accepts this format.

*Step 7* Considering Salhia land2 which has a smaller number of nodes, for reducing calculations, take a snapshot of its status at a moment by considering the GeoJSON represents all of its current data.

*Step 8* Construct tables for computing CQI, PQI, FQI, LS for each node and compare the result with their values in the GeoJSON.

*Step 9* Increase the interpolation grid cellSize to 0.1 km to reduce the grid values; draw the contours together with the interpolation grid values to validate the resulted zones and their boundaries.

*Step 10* Adjust the data sent by Salhia land1 and Salhia land2 such that in certain points of time the events related to the two lands are issued.

#### The validation test1 results

**Figure 17:**
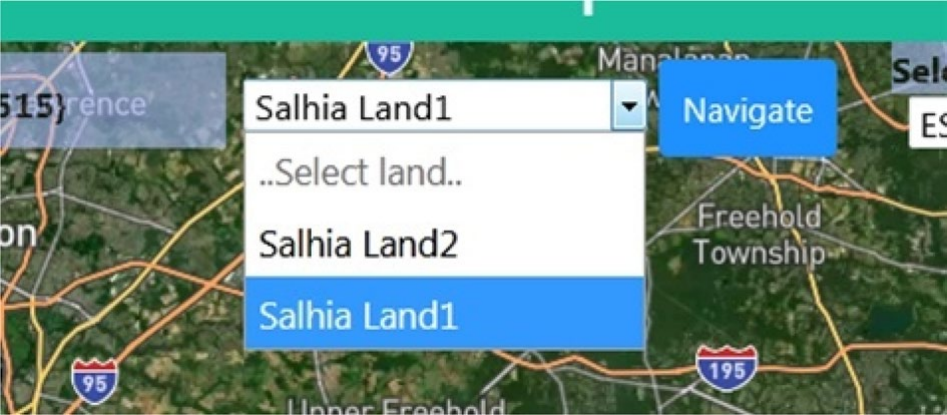
The registered lands list.

After steps 1 and 2, the lands drop down menu is populated automatically with the lands names, Fig. 17.

**Figure 18:**
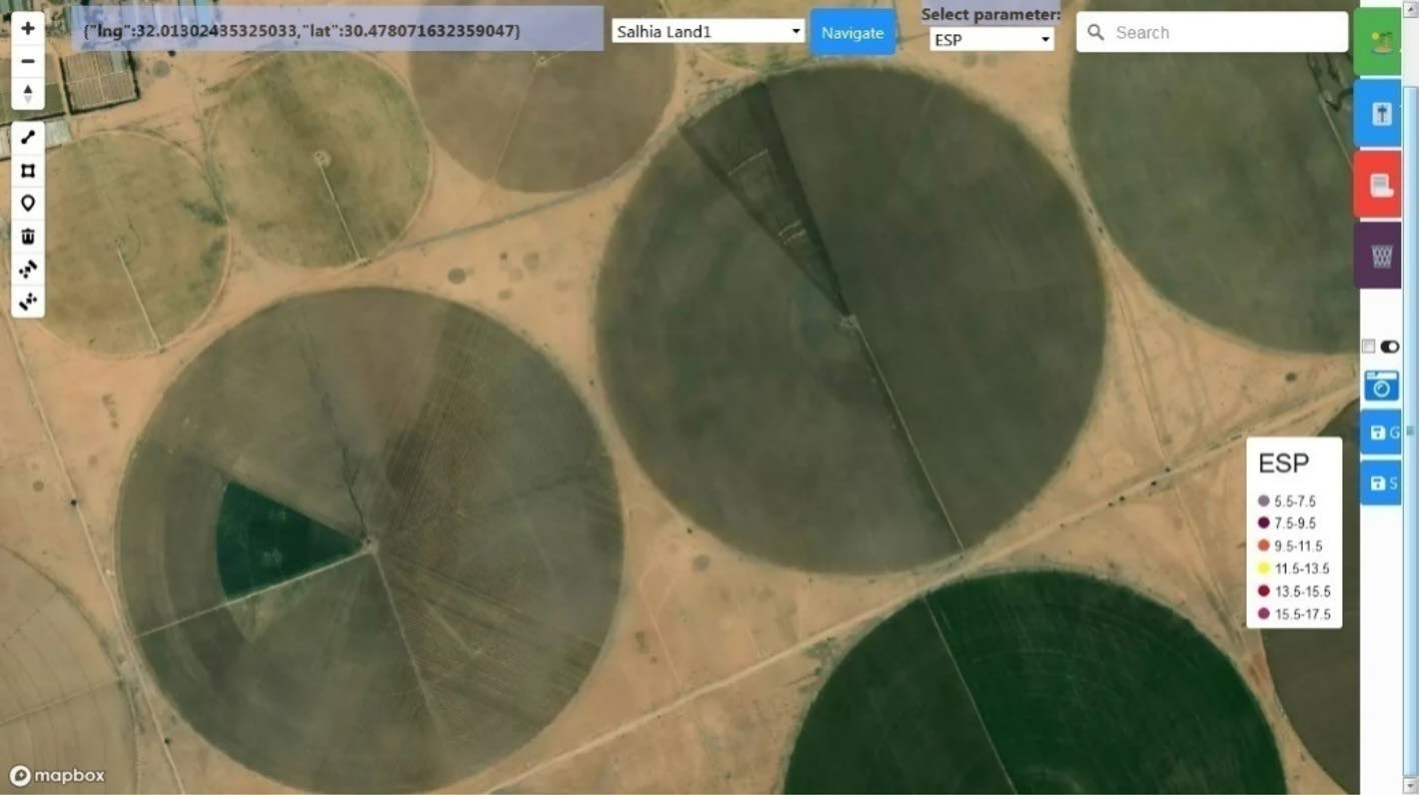
The two adjacent test lands.

We can navigate to them, in Fig. 18, they are the two adjacent lands appear.

The IoT subscriptions and publications were validated by Steps 3 and 4. Just after the first message from a node in Salhia land1 was received, an icon representing its real position appears.

**Figure 19:**
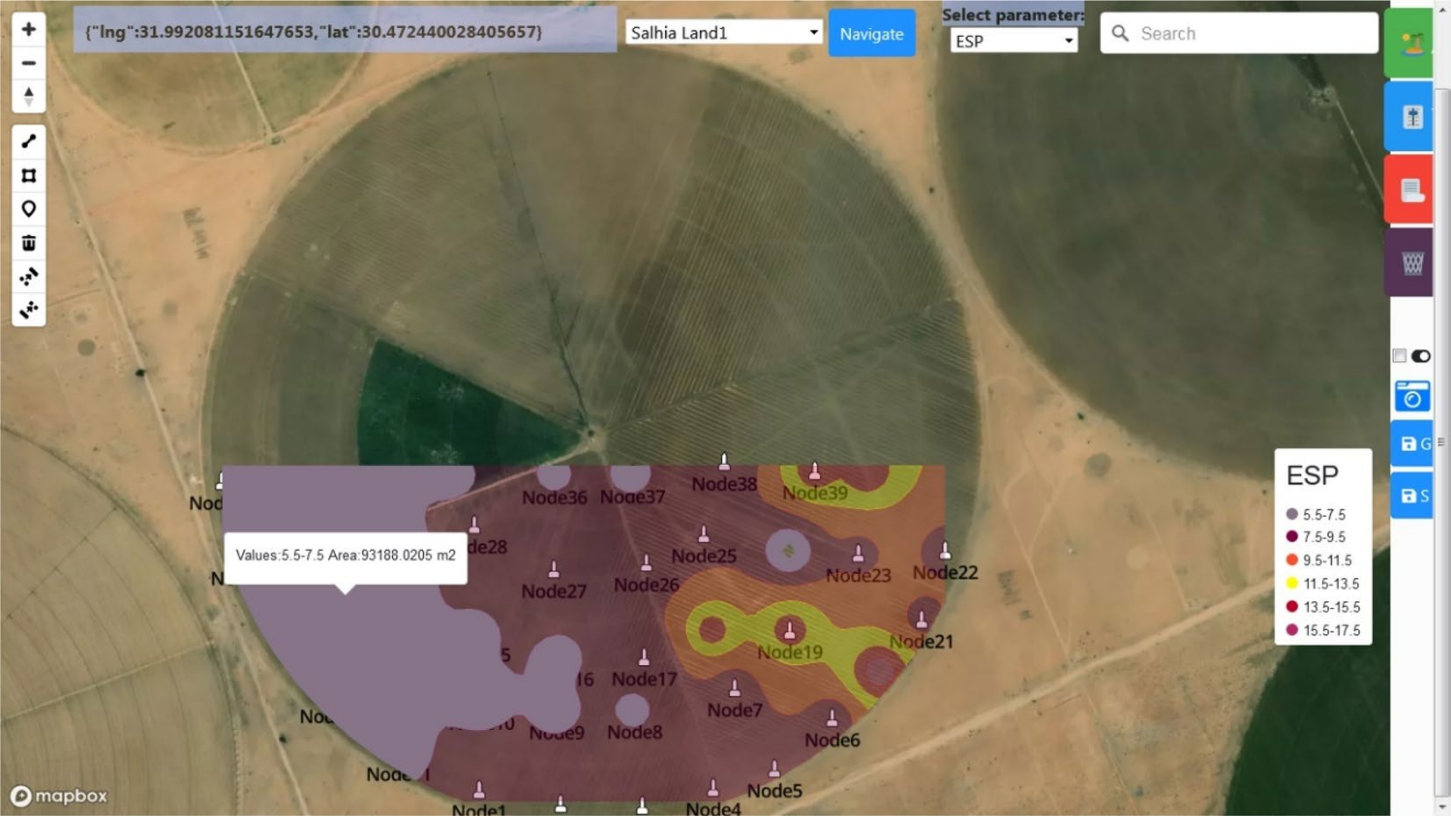
Gradual construction of MZ map while the nodes start sending data.

In the same fashion, the remaining deployed nodes gradually appear, and gradually the zones map is constructed in the region encloses the nodes sent data until the map of the whole land is constructed for the currently selected parameter, see Fig. 19. The zones are drawn with low level opacity colors, when we hover with the mouse cursor on a zone, it is highlighted, the values range it represents and also its area in square meter appear. The zones map that depends on a real-time parameter is continuously updated once a new real-time value is received.

**Figure 20:**
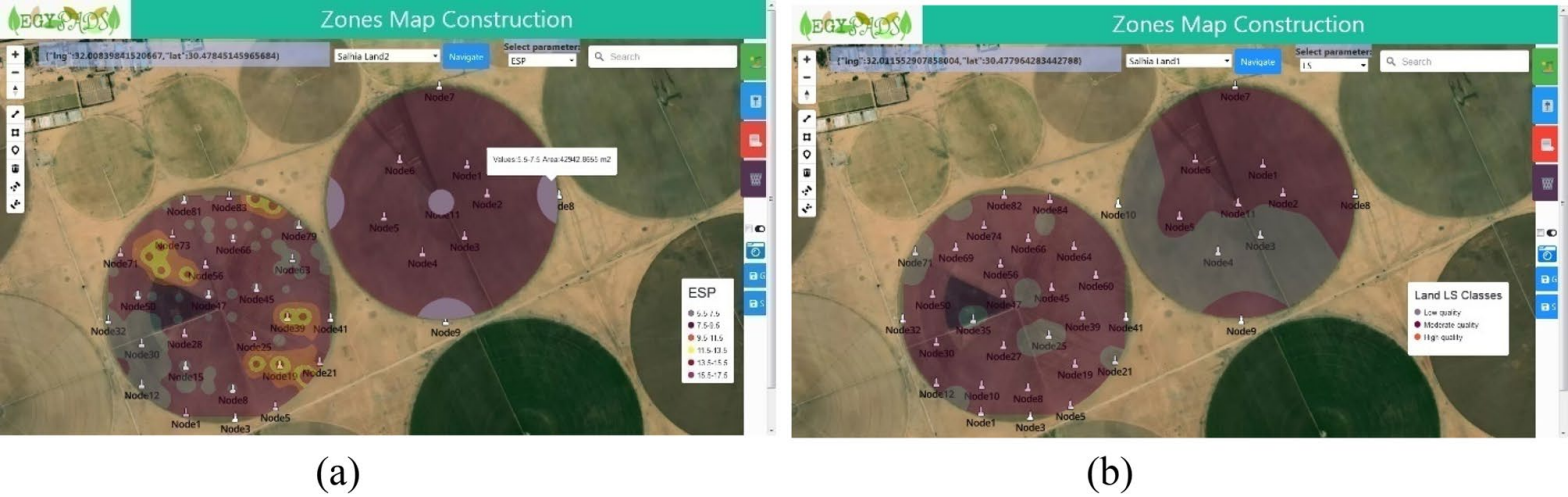
The complete MZ map for the two lands for two parameters, (**a**) the ESP maps, (**b**) LS classes maps.

The same gradual construction happened for Salhia land2 as a result of Step4 until the complete construction. The selected parameter for analysis can be changed, see Fig. 20.

**Figure 21:**
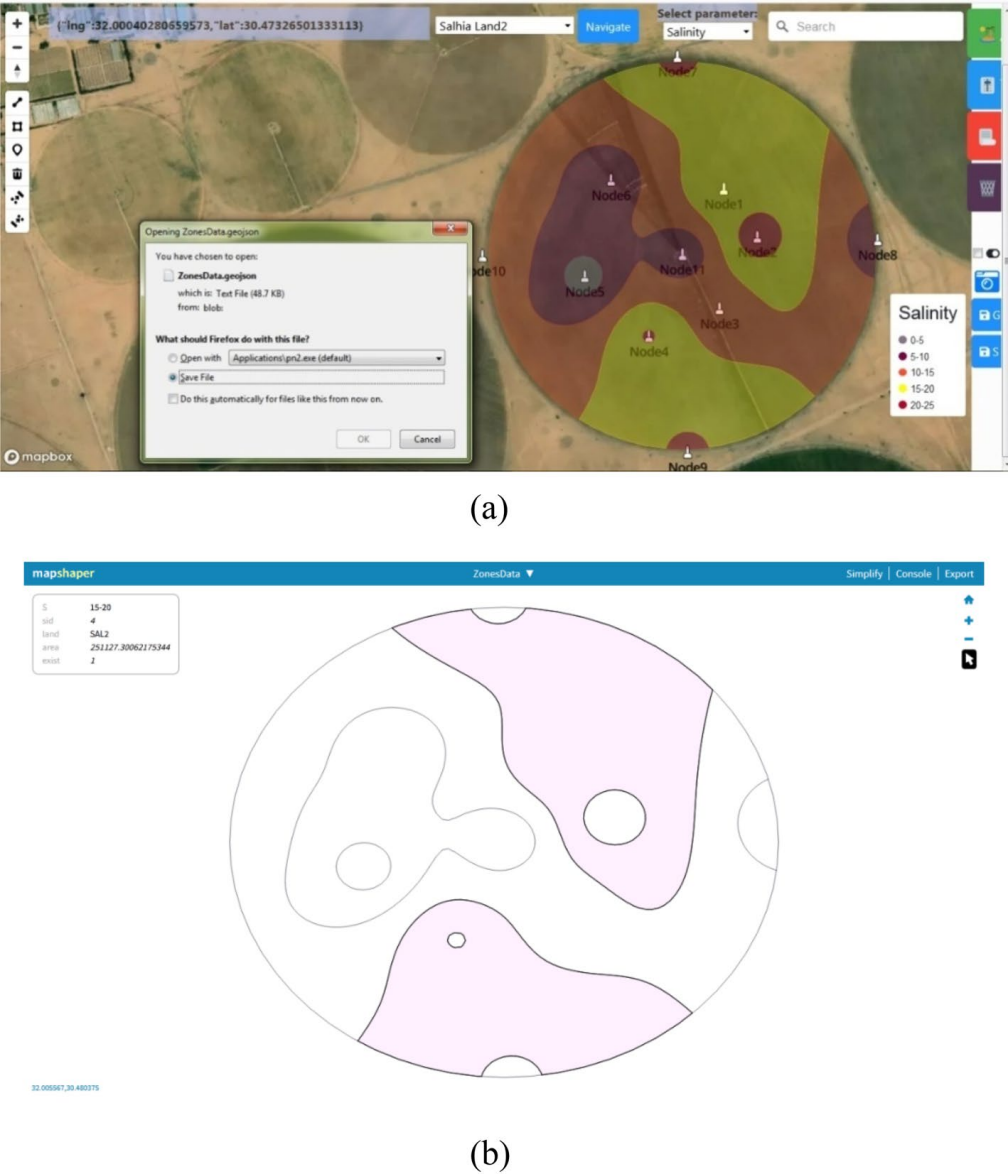
Exporting the MZ map as GeoJSON and Shapefile, (**a**) save map, (**b**) open the saved map using Mapshaper.

According to Step6, a map for Salhia land2 salinity is exported as GeoJSON and then Shapefile, and opened using Mapshaper which is a software for editing GeoJSON and Shapefile. Figure 21a shows the exported map and Fig. 21b shows it in the Mapshaper.

Tables 5 and 6 show the result of Steps 7 and 8. Node1 is randomly selected for the validation calculations. The manually calculated indices are the same as the system-calculated values.

**Table 5:** Node1 factor scores determination.

	N	P	K	OM	Zn	R	T	D	F	Y	HP	G	WHC	S	ESP	CaCO3	PH
Value	140	5	18.2	0.129704	1.47008	101	11	53	0.611565	36.5069	100	1.14213	41.3703	9.575182	8.4	0.65	8.679235
Factor score	1	0.5	0.2	0.2	1	1	0.2	0.8	1	0.5	0.8	0.8	0.8	0.5	1	1	0.2

**Table 6:** Node1 calculations.

	CQI (S_s_ × S_ESP_ × S_CaCO3_ × S_PH_)^1/4^	FQI (S_N_ × S_P_ × S_K_ × S_OM_ × S_Zn_)^1/5^	PQI (S_R_ × S_T_ × S_D_ × S_F_ × S_Y_ × S_HP_ × S_G_ × S_WHC_)^1/8^	LS (CQI × FQI × PQI)^1/3^
Manually computed value	0.56234132519034908039495103977648	0.45730505192732634640270232387125	0.67072577120033136856748879460385	0.55665185954935052194037176247932
System computed value	0.5623413251903491	0.45730505192732634	0.67072577120033136	0.55665185975810261

**Figure 22:**
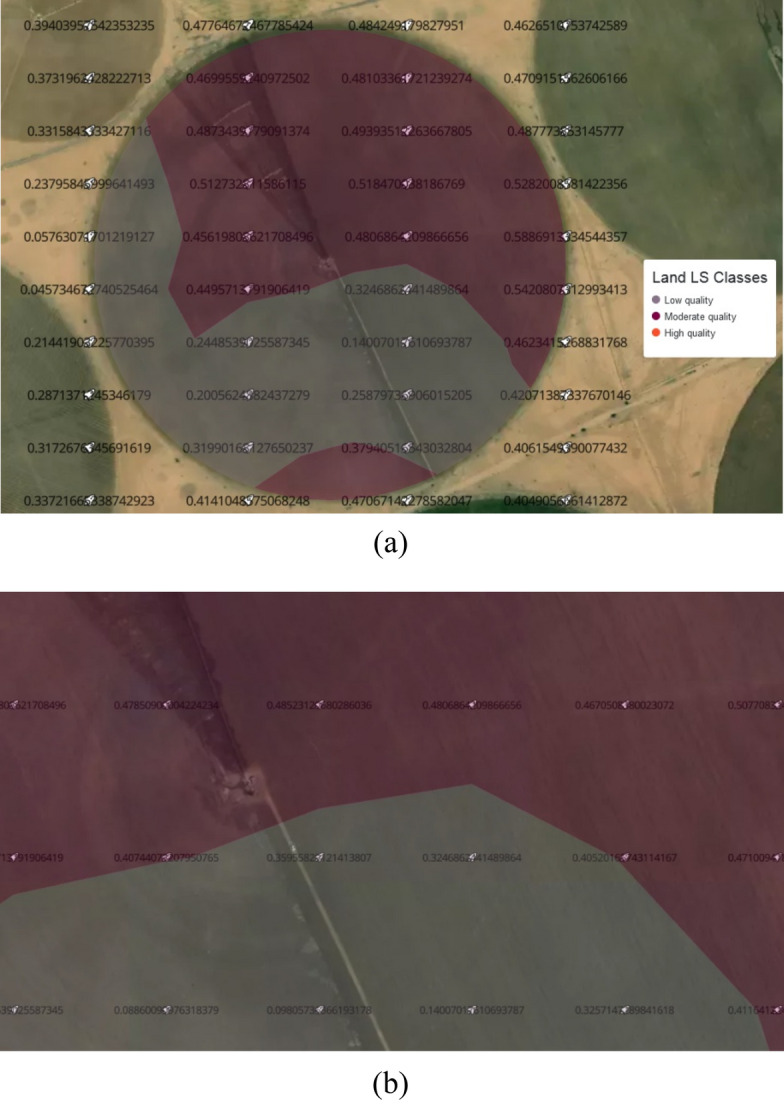
The generated LS interpolation grid and isobands, (**a**) the grid and isobands of the whole area, (**b**) a zoom in specific boundary between two isobands.

The result of Step 9 for the LS index is shown in Fig. 22a where the grid of LS interpolation values that is produced by turf interpolate and the LS classes isobands which are generated by turf isobands are shown. We can zoom in specific boundary to see more values constructing the two isobands [0–0.4] and [0.4–0.7], Fig. 22b.

**Figure 23:**
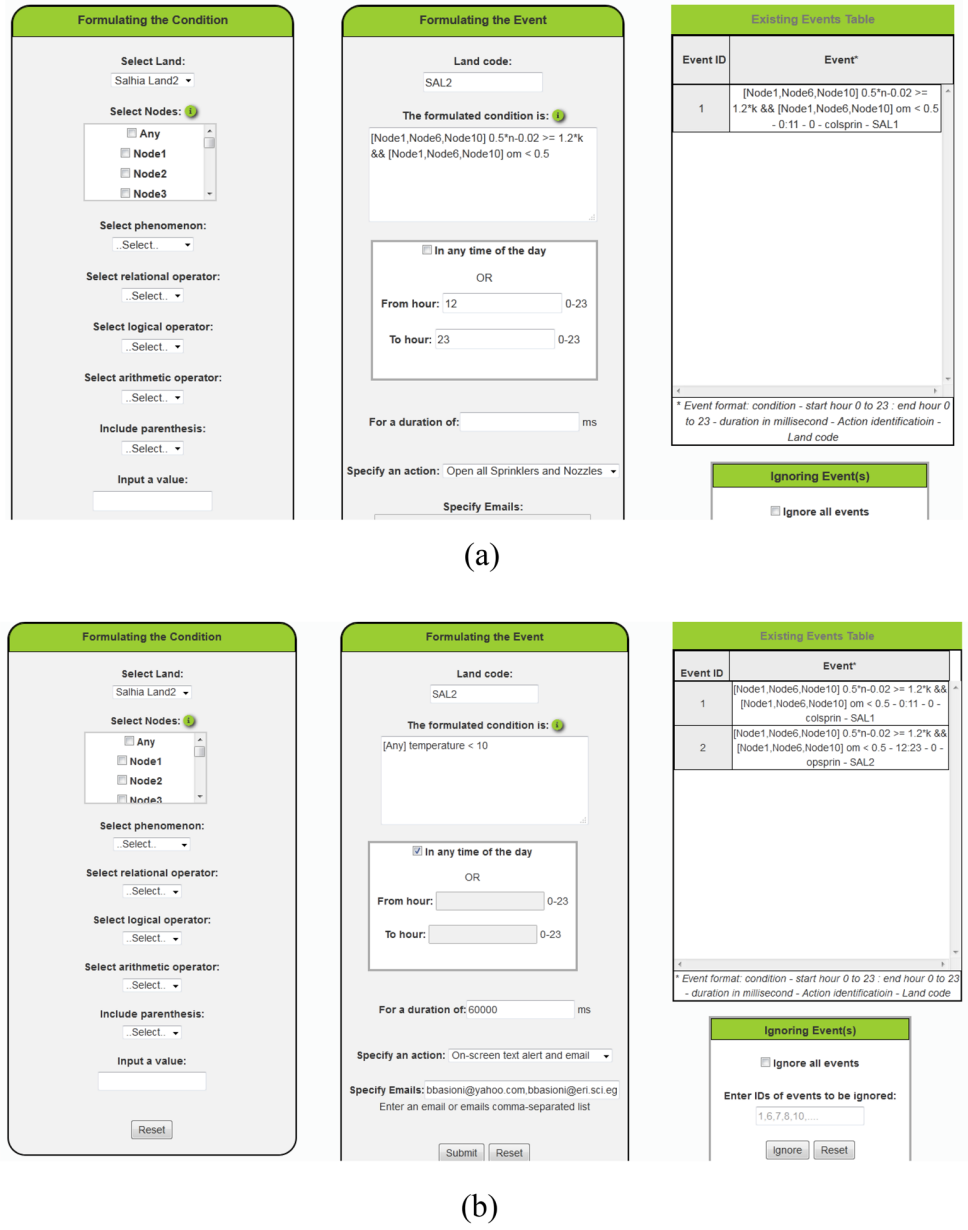
Formulation of the two events required by the validation test to be checked.

For Steps 5 and 10, first, all the existing events were ignored, then the required events were formulated and submitted, Fig. 23 shows the translation of the events required to be tested for Salhia land2.

**Figure 24:**
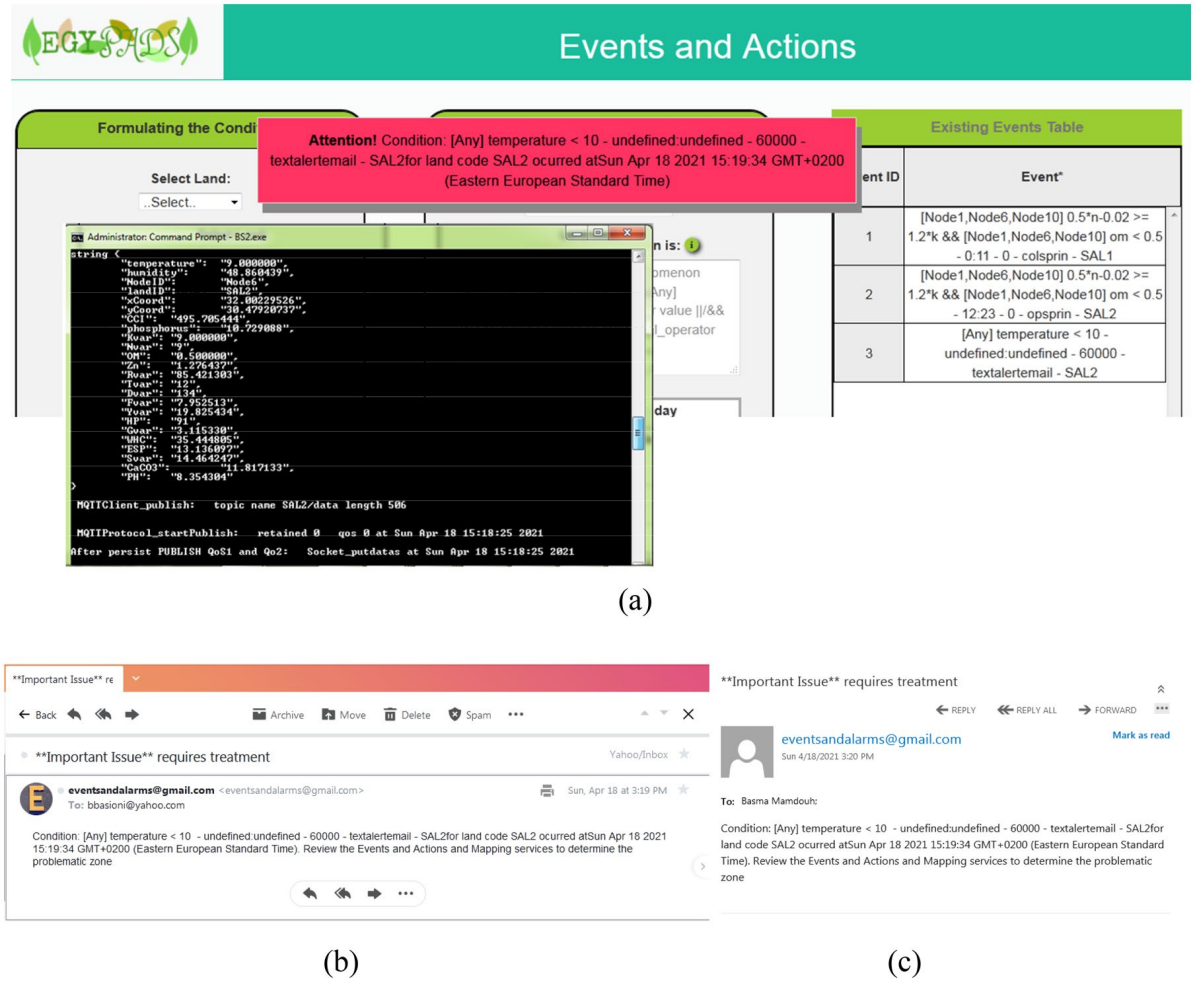
The action of the temperature event takes place, (**a**) an on-screen text alert message, (**b**) email message to the first email, (**c**) email message to the second email.

Node6 in Salhia land2 is selected to fire the temperature event, accordingly its temperature readings are adjusted to take this sequence: {19, 18.5, 9, 9, 9.5, 9.8, 9, 9, 8, 8, ………}. It is evident from this that the third reading fulfills the temperature condition, but the event will fire only if it remains less than 10 for a period of 1 min. The result is shown in Fig. 24, where after the 9 °C temperature value of Node6 was received by the server, the event was fired after that by 1 min and its associated actions were taken: the on-screen text alert message and the email messages to the event's registered email addresses.

**Figure 25:**
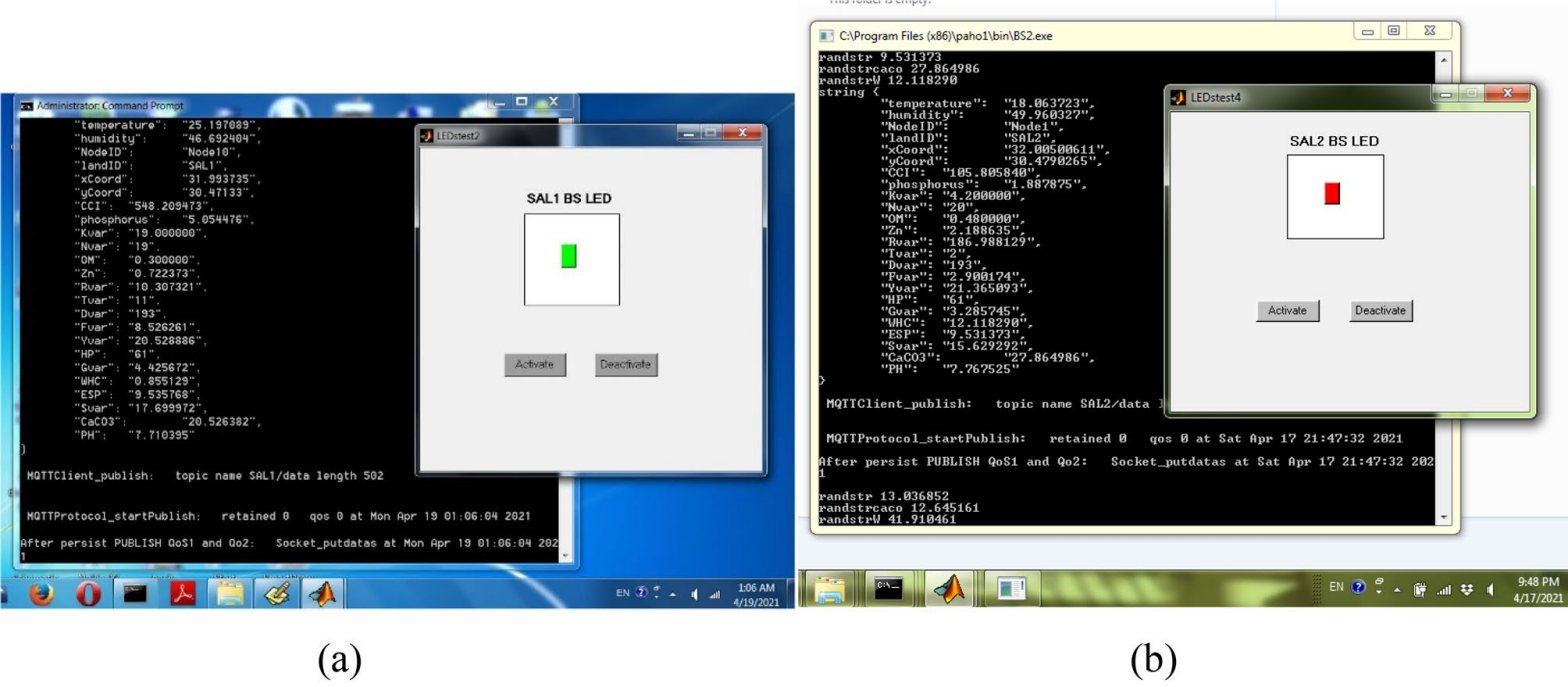
The action of sending commands is fired, (**a**) an Close command for Salhia land1, (**b**) an Open command for Salhia land2.

The condition of the remaining two events for Salhia land1 and Salhia land2 is composed of six sub-conditions. The first three sub-conditions are related to the N and K of the three nodes which are laboratory parameters entered by the excel sheet and their values are adjusted to make these sub-conditions true. The remaining three sub-conditions depend on the value of the OM readings sent by the three nodes; they are adjusted to take these values for Salhia land1: Node1{0.4}, Node6{0.48}, Node10{0.3}, and take this sequence for Salhia land2: Node1{ 0.5, 0.48,0.55, 2, 1.5, ……….}, Node6{ 0.3, 0.47,0.5, 0.55, 0.5, ……….}, Node10{ 0.47, 0.47, 1,0.5,0.5, ……….}. For Salhia land1, just after the reading of Node10 was received at 1:06, the second three sub-conditions were satisfied and the command Close was sent to Salhia land1 base station where it blinked the LED with the green color to indicate the reception of Close command, see Fig. 25a. For Salhia land2, the first three readings of the three nodes satisfies only the sub-conditions of Node6 and Node10, just after Node1 sent its second reading at 21:47:32, its sub-conditions also became true, therefore an Open command was sent to Salhia land2 base station where it blinked the LED with the red color to indicate the reception of Open command, see Fig. 25b.

#### The validation test2 scenario

**Figure 26:**
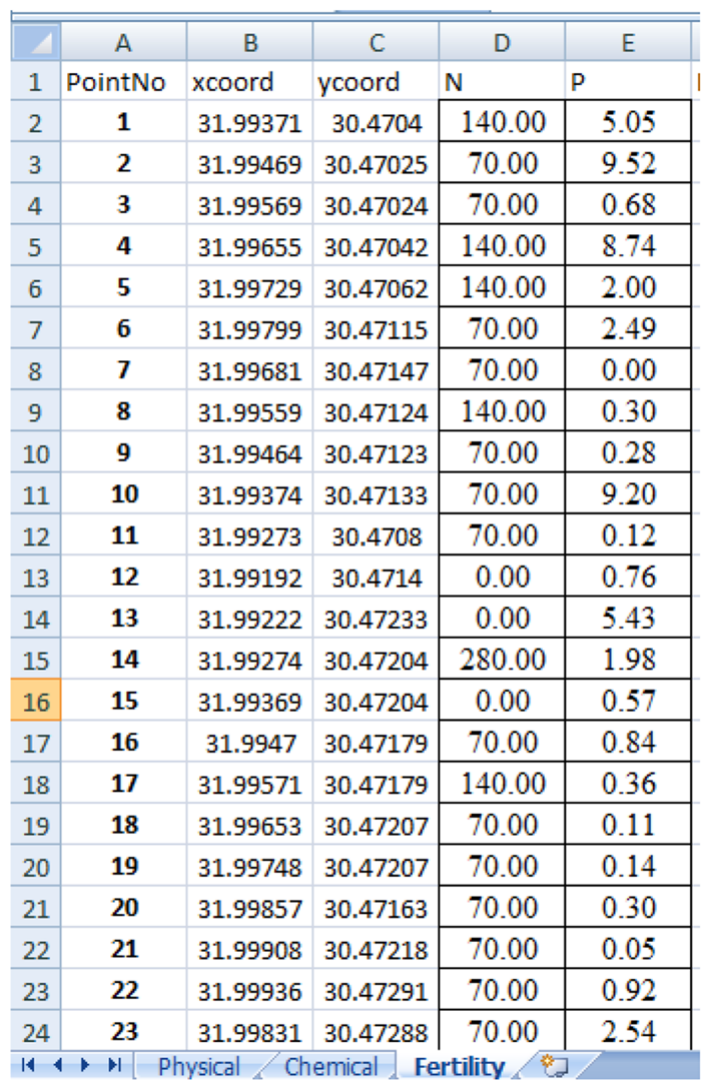
The acceptable format of the excel sheet data.

This test is related to the land of the field trips, Salhia land1 in test1 with the same settings of system parameters. This test aims at producing maps of parameters using both the traditional method and EGYPADS. The function of EGYPADS Mapping service, which allows for entering all nodes data manually through a specific format excel sheet, is employed in this test. The 85 sample values of three parameters (P, N, and PH), which were measured/estimated and analyzed in the field trips, were entered into EGYPADS representing nodes readings, and their spatial analysis maps were generated. The acceptable format of the excel sheet is shown in Fig. 26.

#### The validation test2 results

**Figure 27:**
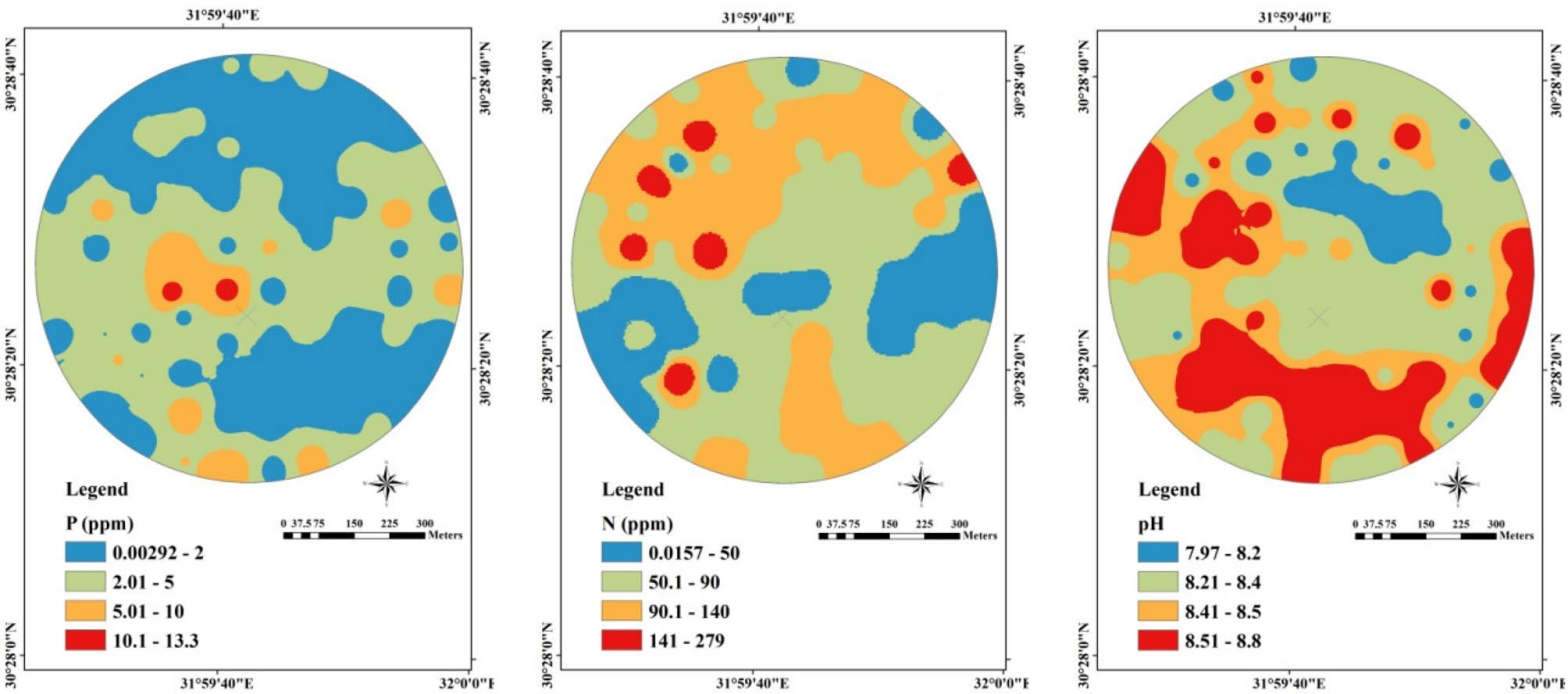
The GIS-generated maps.

**Figure 28:**
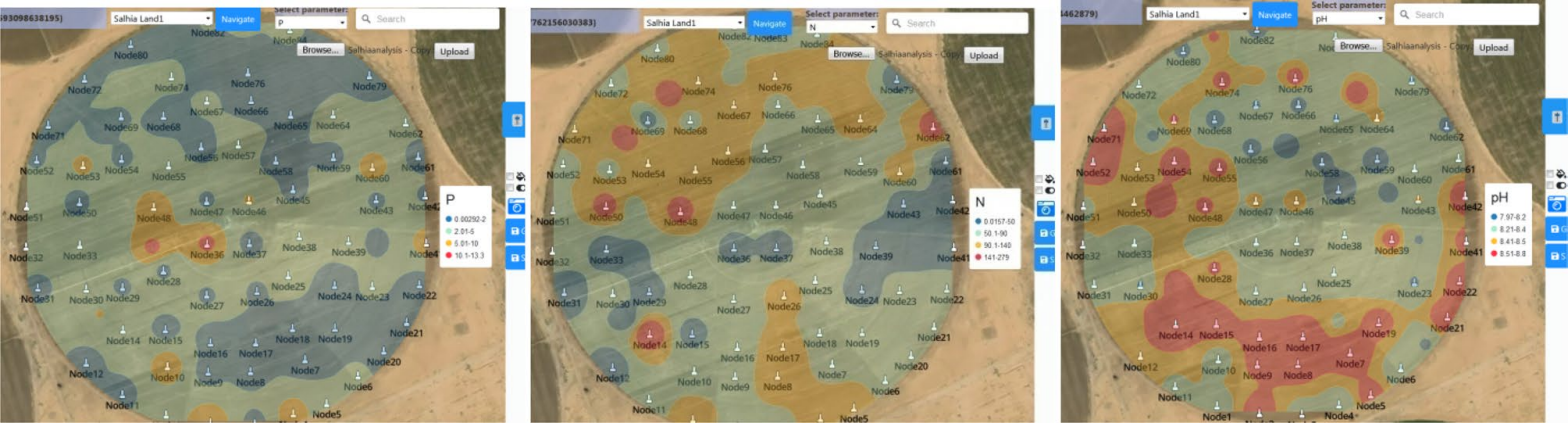
The EGYPADS-generated maps.

Figure 27 and 28 show the management zones maps of P, N, and PH generated from the GIS used in the traditional method and the EGYPADS, respectively. It is noticed that, in case of EGYPADS, there are small areas at the four horizontal and vertical borders of the land don't belong to any zone. This is because that EGYPADS requires putting at least one sensor node at each of the farthest four ends of the land in order to divide the whole area of the region into management zones, and this not satisfied by the grid system used to collect the data in the field visits. This causes the total area of the EGYPADS management zones to be less than GIS zones total area by about 5.6%. Also, it is noticed that EGYPADS produces the same zones of the parameters with slight difference in zones shape and area accordingly.

The values of zones areas using the two methods are given in Table 7. Generally, there is a reduction in the EGYPADS zones areas than the GIS due to the just mentioned reason and of course due to different implementation of methods in the two approaches. The average difference in the zones area is estimated to be approximately 24.77%.

**Table 7:** Management zones areas resulted from the LSA approaches.

Parameter	Zone	Zone area using the traditional method GIS (m^2^)	Zone area using EGYPADS (m^2^)	Average difference in area between the two programs implementations (%)
P	0.00292–2	288,721.8037	240,079.2785	26.8155
	2.01–5	310,225.9135	339,900.2504	
	5.01–10	48,919.3858	32,627.2506	
	10.1–13.3	3247.0045	1703.2153	
N	0.0157–50	127,774.6886	84,738.1362	20.58183
	50.1–90	286,474.0909	315,475.6464	
	90.1–140	216,009.3472	199,652.9413	
	141–279	20,917.1248	14,443.271	
PH	7.97–8.2	53,173.6616	22,911.9268	26.91618
	8.21–8.4	302,924.5794	319,275.324	
	8.41–8.5	141,388.2681	164,037.0346	
	8.51–8.8	152,317.3301	107,631.8299	

#### The validation test3

**Figure 29:**
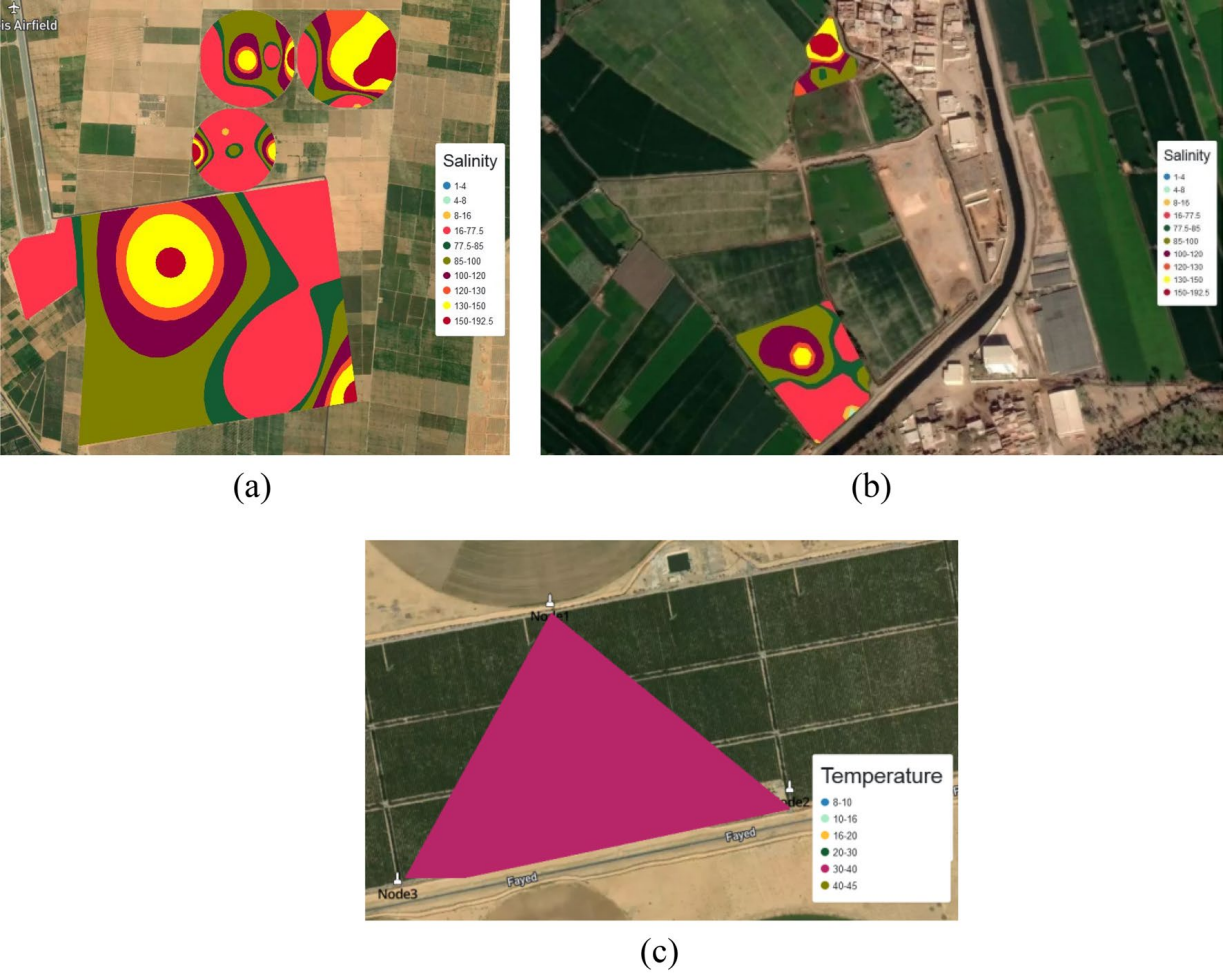
Different lands with different characteristics registered in the system, (**a**) examples of four lands of different area and geometry being monitored concurrently, (**b**) two other lands being monitored concurrently in different location, (**c**) another monitored land receiving data from real base station.

This test validates the ability of the system to support more than one land with different geometry, area, location, and number of nodes. Different lands satisfy these characteristics were registered in the system. Figure 29, shows some of the lands, including the land in Fig. 29c which receives the three nodes reading from the real base station. Since the three sensor nodes don't have GPS, certain coordinates were assigned to them. The temperature readings of the three nodes at the test time had very little variation, all of their readings fell in the 30–40 temperature range. From the test it is concluded that the lower the rates of maps update and data transmission, the more we can register lands and prevent the overlap between zones.

## Discussion

LSA for land-use planning and management is the core of the implemented IoT-based PA system use case. It is a multi-criteria decision support problem, and the research in this paper includes the conventional method used to solve this problem besides the implemented automated DSS. The conventional method requires performing a field trip may include travels to distant places, a considerable number of labors to take the readings in the predefined sampling points and record them, using PC-based programs for data analysis which may not be user friendly and require skilled person to use it. This is for one-time field sampling which doesn't represent the temporal distribution of phenomena and entails other trips. The implemented system allows sensing and actuation nodes to work as the data collection labors distributed in the field in a way that satisfies the required spatial distribution of the phenomena, and remain in the field sending data from their places in real-time with any required rate. All received data is stored by the system, and just after the data reception, it automatically performs the spatial analysis and displays the results; in this way if a critical condition occurs to the field, it will be detected early. The system doesn't require its user to do specialized commands. EGYPADS provides a modernization of the LSA activity.

## Conclusion and future work

Proper agricultural management requires evaluating different soil properties. Also, the characteristics of the soil differ from one site to another within the same farm. Real-time monitoring of soil properties especially, moisture and temperature are important for the success of the agricultural process. The proposed system, EGYPADS, allows for continuous monitoring of soil properties, and utilizes this in building a novel feature gives a facility to classify the farm into different zones for proper management in real-time as well as from historical data with high temporal resolution. To the best of our knowledge, this is the first smart agriculture system that automates the practice of land suitability assessment in order to facilitate it and increase its efficiency in terms of accuracy of decision-making, need for manpower, time and effort wasted on repeated field visits. As a smart agriculture application, EGYPADS is designed to take into account the important smart agriculture services, adding new services, and improving the services functionality. EGYPADS has the advantages of providing the facility to enter the data manually along with its inherent feature of reading directly from field sensor nodes. Although the proposed software gave reasonable results for soil management zones of the potato crops, it needs to include some functions regarding the fertilization and vegetation growth, in addition, to use for other crops and some factors that will be considered in the second version.

## Data Availability

Ethical, legal, and commercial restrictions apply to the availability of the data support the findings of this study, which were used under license for this study. Data are available from the authors, after the permission of the funding entity and the study area owner, upon reasonable request.
